# Fibroblast-derived osteoglycin promotes epithelial cell repair

**DOI:** 10.1038/s41536-025-00404-3

**Published:** 2025-03-25

**Authors:** Luke van der Koog, Manon E. Woest, Iris C. Gorter, Vicky Verschut, Robin A. B. Elferink, Annet B. Zuidhof, Dyan F. Nugraha, Maunick L. Koloko Ngassie, Sophie I. T. Bos, Deepesh Dhakad, Justina C. Wolters, Peter L. Horvatovich, Y. S. Prakash, Wim Timens, Önder A. Yildirim, Corry-Anke Brandsma, Henderik W. Frijlink, Anika Nagelkerke, Reinoud Gosens

**Affiliations:** 1 Department of Molecular Pharmacology, Groningen Research Institute of Pharmacy, University of Groningen, Groningen, The Netherlands; 2 Groningen Research Institute for Asthma and COPD, University Medical Center Groningen, Groningen, The Netherlands; 3 Aquilo BV, Groningen, The Netherlands; 4 Department of Pathology and Medical Biology, University Medical Center Groningen, Groningen, The Netherlands; 5 Department of Anesthesiology and Perioperative Medicine, Mayo Clinic, Rochester, MN USA; 6 Institute of Lung Health and Immunity (LHI), Comprehensive Pneumology Center (CPC), Helmholtz Munich, Member of the German Center for Lung Research (DZL), Munich, Germany; 7 Department of Pediatrics, University Medical Center Groningen, Groningen, The Netherlands; 8 Department of Analytical Biochemistry, Groningen Research Institute of Pharmacy, University of Groningen, Groningen, The Netherlands; 9 Institute of Experimental Pneumology, University Hospital, Ludwig-Maximilians University, Munich, Germany; 10 Department of Pharmaceutical Technology and Biopharmacy, Groningen Research Institute of Pharmacy, University of Groningen, Groningen, The Netherlands; 11 Department of Pharmaceutical Analysis, Groningen Research Institute of Pharmacy, University of Groningen, Groningen, The Netherlands

**Keywords:** Respiratory tract diseases, Therapeutics, Translational research

## Abstract

There is an urgent need for innovative therapies targeting defective epithelial repair in chronic diseases like COPD. The mesenchymal niche is a critical regulator in epithelial stem cell activation, suggesting that their secreted factors are possible potent drug targets. Utilizing a proteomics-guided drug discovery strategy, we explored the lung fibroblast secretome to uncover impactful drug targets. Our lung organoid assays identified several regenerative ligands, with osteoglycin (OGN) showing the most profound effects. Transcriptomic analyses revealed that OGN enhances alveolar progenitor differentiation, detoxifies reactive oxygen species, and strengthens fibroblast-epithelial crosstalk. OGN expression was diminished in COPD patients and smoke-exposed mice. An active fragment of OGN (leucine-rich repeat regions 4–7) replicated full-length OGN’s regenerative effects, significantly ameliorating elastase-induced lung injury in lung slices and improving lung function in vivo. These findings highlight OGN as a pivotal secreted protein for alveolar epithelial repair, positioning its active fragment as a promising therapeutic for COPD.

## Introduction

There is a growing interest in therapeutic strategies aimed at restoring defective tissue repair in chronic diseases to cure or slow down the decline in organ function. In the lung, regenerative medicine has primarily focused on transplantation, tissue engineering, stem or progenitor cell therapy, or a combination of these approaches^1,2^. A regenerative pharmacological approach holds considerable promise due to its potential for large-scale application and early intervention in the disease process^3^. Currently, no approved drugs support lung tissue repair.

Within respiratory medicine, chronic obstructive pulmonary disease (COPD) is the most common chronic lung disease with a need for better therapies^2,4^. The underlying pathogenetic mechanism of COPD involves a complex interplay between enhanced destruction of alveolar septa and a diminished capacity of alveolar epithelial progenitor cells to support epithelial tissue repair^5–7^. The alveolar epithelium comprises alveolar type (AT)1 cells, which facilitate gas exchange, and AT2 cells, which secrete pulmonary surfactants and possess stem cell-like characteristics essential for lung repair^8,9^. Cell types within the alveolar niche, such as distal lung fibroblasts, provide structural support, secrete extracellular matrix, and provide paracrine molecular cues that direct proper proliferation and differentiation of the alveolar epithelium^9–13^.

In COPD, aberrant fibroblast-epithelial signaling contributes to remodeling and impaired epithelial repair^14–18^. Recent findings show that the progenitor cell niche is a driver of defective epithelial repair and that targeting the niche pharmacologically is feasible^3,19^. The importance of this process is illustrated by distal lung alveolar epithelial progenitors, which differentiate into AT1 and 2 cells in organoids. These require mesenchymal support cells and only form if these mesenchymal cells are in contact with epithelial progenitors^9,17^. Additionally, we have previously demonstrated that lung fibroblast-derived secretome ameliorated lung tissue injury^20^. Exposure of these mesenchymal cells to cytokines and factors (e.g., transforming growth factor (TGF)-β, cigarette smoke (CS)) that drive the disease process in COPD strongly impairs their promotive role^17,18,21^.

Lung fibroblasts support alveolar epithelial regeneration through several mechanisms, including paracrine signaling, by releasing extracellular vesicles (EVs) and soluble factors (SFs)^22^. Here, we aimed to identify factors in the lung fibroblast secretome with regenerative potential for therapeutic development. Using a proteomics-guided drug discovery strategy, we identified osteoglycin (OGN) and its active fragment to have significant potential in supporting alveolar epithelial repair. We also demonstrate reduced expression of OGN in lung tissue from current smokers and patients with smoking-associated COPD. Additionally, we demonstrate that treatment with OGN and its active fragment ameliorates elastase-induced injury in precision-cut lung slices (PCLS) and restores lung function in mice.

## Results

### Lung fibroblast-derived EVs and SFs support alveolar epithelial growth

**Fig. 1:**
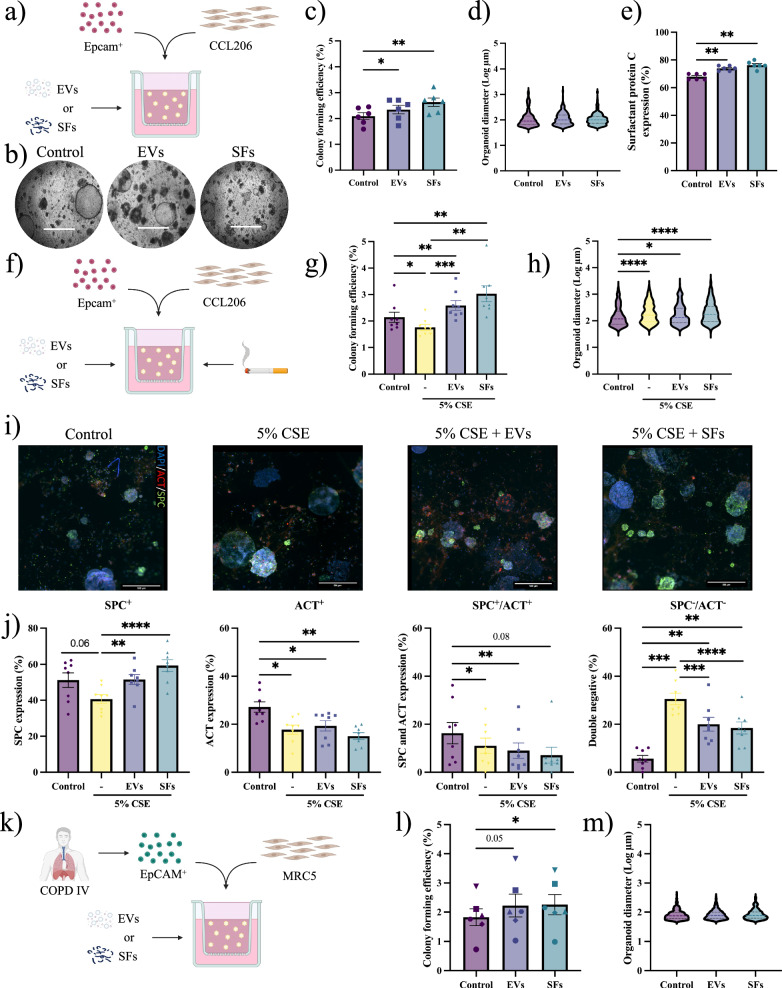
Lung fibroblast-derived EVs and SFs support alveolar organoid formation. **a** Schematic of in vitro murine organoid design treated with EVs or SFs. **b** Brightfield images of murine lung organoids on day 14 (scale = 700 µm). **c** CFE of murine organoids (mean ± SEM, N = 6, one-way ANOVA, Dunnett test). **d** Log of murine organoid diameter (median, N = 6, Kolmogorov-Smirnov test, α = 0.0025). **e** Immunohistochemistry quantification for SPC in murine organoids (mean ± SEM, N = 6, one-way ANOVA, Dunnett test). **f** Schematic of in vitro CSE-exposed murine organoid design. **g** CFE of CSE-exposed murine organoids (mean ± SEM, N = 8, one-way ANOVA, Dunnett test). **h** Log of CSE-exposed murine organoid diameter (median, N = 8, Kolmogorov-Smirnov test, α = 0.0167). **i** Immunofluorescence images of organoids for airway-type (ACT, red), alveolar-type (SPC, green), and DAPI (nuclei, blue) (scale = 500 µm). **j** Immunohistochemistry quantification for SPC^+^, ACT^+^, SPC^+^/ACT^+^, and SPC^-^/ACT^-^ organoids (mean ± SEM, N = 6, one-way ANOVA, Dunnett test). **k** Schematic of in vitro human organoid design. **l** CFE of human organoids (mean ± SEM, N = 6, one-way ANOVA, Dunnett test). **m** Log of human organoid diameter (median, N = 6, Kolmogorov-Smirnov test, α = 0.025). Statistically significant comparisons: *p < 0.05, **p < 0.01, ***p < 0.001, ****p < 0.0001.

To assess the regenerative potential of the secretome of human lung fibroblasts, we used an established in vitro murine lung organoid model^3^. This organoid model of primary adult lung epithelial tissue closely recapitulates epithelial regeneration in the lung, mimicking the initial activation of lung progenitor cells, their subsequent proliferation, and the differentiation into specialized epithelial cells. Organoid number reflects the activation of epithelial progenitor cells to initiate division and form organoids, while organoid size provides insights into both proliferation and differences in phenotype^3^. We assessed whether fibroblasts exert their preventive function in a paracrine manner through the secretion of extracellular vesicles and soluble factors (Fig. 1a). MRC5 human lung fibroblast-derived EV-enriched fractions (EVs) and SFs-enriched fractions (SFs) were purified using a combination of ultrafiltration and size exclusion chromatography, and previously characterized according to MISEV guidelines^20,23^. Morphological assessment of EV-enriched fractions revealed intact, spherical vesicles with a diameter of around 110 nm (Fig. S1). The colony forming efficiency (CFE) of organoids established by co-culturing murine lung tissue-derived CD31^-^/CD45^-^/Epcam^+^ cells and CCL206 fibroblasts was evaluated. Freshly isolated CD31^-^/CD45^-^/Epcam^+^ cells are mainly composed of AT2 cells (around 80–85%) as we have demonstrated previously using FACS and RNAseq analysis^17,24,25^. The CFE was significantly increased by treating cultures with EVs (10^9^ particles/mL, 50,000 particles per cell) or SFs (30 µg/mL) (Fig. 1a–c), without affecting organoid size (Fig. 1d). In addition, EV- or SF-treatment increased organoid formation in a concentration-dependent manner (Fig. S2a–d). Interestingly, reducing the number of fibroblasts yielded significantly fewer murine organoids, underscoring the supportive function of fibroblasts in epithelial organoid formation (Fig. S2e–f). We compared treatment effects with EVs or SFs in the presence of varying fibroblast numbers: 10,000, 5000, or 2500 CCL206 fibroblasts (Fig. S2g–j). As the number of fibroblasts decreased, both treatments with EVs (10^9^ particles/mL) or SFs (30 µg/mL) exhibited a more pronounced increase in CFE (Fig. S1g–j). In contrast, culturing Epcam^+^ cells in the absence of fibroblasts resulted in no organoid formation, even when treated with fibroblast-derived EVs or SFs, suggesting that the presence of fibroblasts is essential for supporting organoid development^17^. To specifically analyze the impact of treatment with EVs or SFs on organoids derived from alveolar epithelial progenitors, we used immunofluorescence staining to distinguish between airway-type organoids (ACT^+^) and alveolar-type organoids (SPC^+^) (Fig. S2k). This analysis revealed that alveolar epithelial progenitors were more prone to induce CFE to treatment with EVs or SFs compared to control, yielding significantly more SPC^+^ organoids (Fig. 1e) without affecting the number of ACT^+^ organoids (Fig. S2l).

During COPD development, the alveolar epithelial cells are exposed to a hostile microenvironment, which changes their composition, phenotype, and responses to molecules. To simulate this hostile microenvironment in our organoid model, we exposed organoid cultures to 5% CS extract (CSE; Fig. 1f), which yielded, in accordance with previous findings, a significantly lower organoid number (Fig. 1g) and increased organoid diameter (Fig. 1h)^3^. Concurrent treatment with either EVs or SFs was able to counteract the negative effects of CSE in respect of CFE number. In contrast, organoid size was increased compared to control (Fig. 1g, h). Immunofluorescence staining revealed that the number of SPC^+^ and ACT^+^ organoids decreased in the presence of 5% CSE (Fig. 1i, j), and the proportion of organoids negative for SPC and ACT increased (Fig. 1j). Treatment with EVs and SFs, however, increased the number of SPC^+^ organoids (Fig. 1j) and lowered the number of double-negative (SPC^-^/ACT^-^) (Fig. 1j). For a translational perspective, we further investigated lung fibroblast-derived EVs and SFs on human organoid formation derived from EpCAM^+^ epithelial cells isolated from COPD IV patient lung tissue (Fig. 1k). EV- and SF-treatment increased the CFE of human organoids, whereas diameter was unaffected by both treatments (Fig. 1l, m).

### A proteomics-guided drug discovery strategy identifies osteoglycin to induce organoid formation

**Fig. 2:**
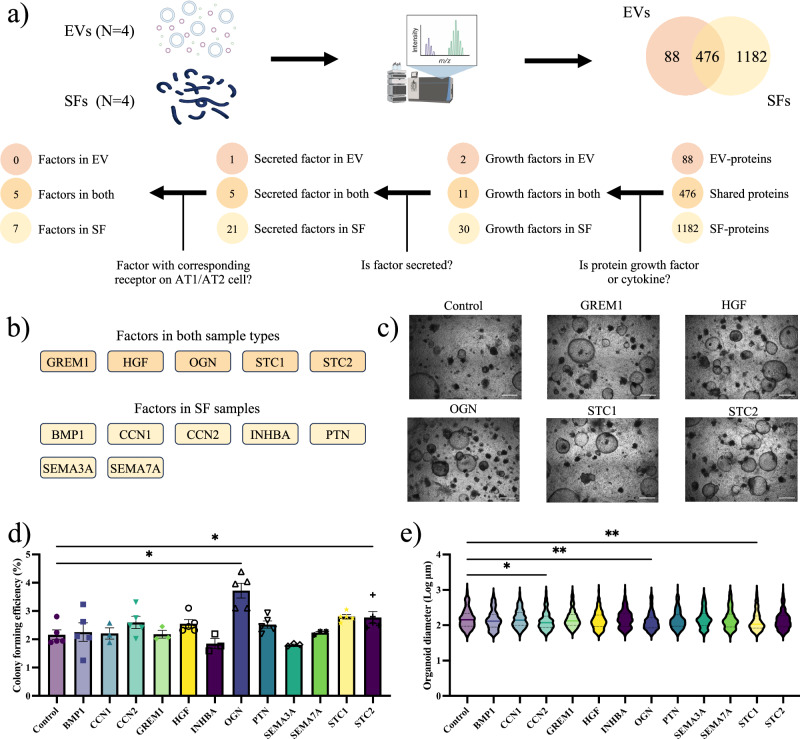
A proteomics-guided drug discovery strategy identifies osteoglycin to induce organoid formation. **a** Schematic outline of the proteomics-guided drug discovery strategy using human lung fibroblasts (MRC5). **b** Overview of included factors in the secretome of human lung fibroblasts. **c** Representative brightfield image of murine organoid cultures for proteomics-guided drug discovery strategy with factors in both EVs and SFs. **d** CFE of murine organoids in proteomics-guided drug discovery strategy (mean ± SEM, N = 3–5, paired one-way ANOVA, Dunnett test). **e** Log of murine organoid diameter in proteomics-guided drug discovery strategy (median, N = 3–5, Kolmogorov–Smirnov test, Bonferroni correction: α = 0.004). Statistically significant comparisons: *p < 0.05, **p < 0.01.

We used a proteomics-guided drug discovery strategy (Fig. 2a) to identify potential therapeutic factors within the fibroblast secretome that may restore defective lung repair. Analysis of lung fibroblast-derived EVs and SFs (N = 4) revealed 1262 proteins and 2090 proteins that were identified in at least one of the replicates, respectively. To demonstrate the nature of the EVs, we analyzed the presence of proteins stipulated following the MISEV guidelines^23^. EVs showed enrichment of transmembrane and GPI-anchored proteins associated with EVs, in contrast to the relatively low abundance in SFs (Supplementary Data 1). Additionally, CD90, a marker for mesenchymal stromal cells, was present in all EV samples. In summary, the proteomic analysis collectively demonstrates an enrichment of EV-associated proteins in EVs when compared to SFs.

For subsequent analysis, we focused on 564 EV-proteins and 1658 SF-proteins that were consistently present in all four replicates (Fig. 2a). Notably, 476 of these proteins were found in both the EVs and SFs (Fig. 2a, Supplementary Data 2). Since growth factors and cytokines are known to affect epithelial progenitor cell behavior strongly, we analyzed the number of growth factors and cytokines using the Gene Set Enrichment Analysis tool (www.gsea-msigdb.org) (Fig. 2a). We identified a total of 43 growth factors, of which two factors were exclusively present in EVs, 30 factors in SFs, and 11 factors in both (Fig. 2a). Since our focus was on paracrine interactions, we narrowed down the number of candidates based on predictions for secretion of the factors using Signal IP (https://services.healthtech.dtu.dk/services/SignalP-6.0/) and Phobius (https://phobius.sbc.su.se/), resulting in a total of 32 secreted factors (Fig. 2a, Supplementary Data 3). Lastly, we investigated whether the secreted factor interacts with a receptor expressed in the target recipient cells (AT1 and AT2 cells) (Fig. 2a). In total, we identified twelve factors for evaluation in lung organoids: seven factors within the SF-protein group and five factors present in both sample types (Fig. 2a, b, Supplementary Data 3).

Using murine lung organoid cultures, we assessed the efficacy of these factors in inducing organoid formation (Fig. 2c, d). All information about the recombinant factors used in this study is provided in Supplementary Data 4. Stanniocalcin (STC)-2 increased organoid numbers without affecting size, while bone morphogenetic (BMP)1, hepatocyte growth factor (HGF), pleiotrophin (PTN), and STC1 tended to increase CFE. CCN2 and STC1 significantly reduced organoid size (Fig. 2e). Notably, osteoglycin (OGN) emerged as the most promising factor identified in the secretome of lung fibroblasts, inducing additional organoid formation by approximately 75% compared to controls (Fig. 2c, d). Interestingly, OGN was present in both EVs and SFs (Fig. 2b). Given our previous observations of the supportive effects of both EVs and SFs on alveolar organoid formation (Fig. 1), we decided to further explore the potential of OGN as a therapeutic candidate for lung repair.

### Osteoglycin expression is reduced in response to cigarette smoke

**Fig. 3:**
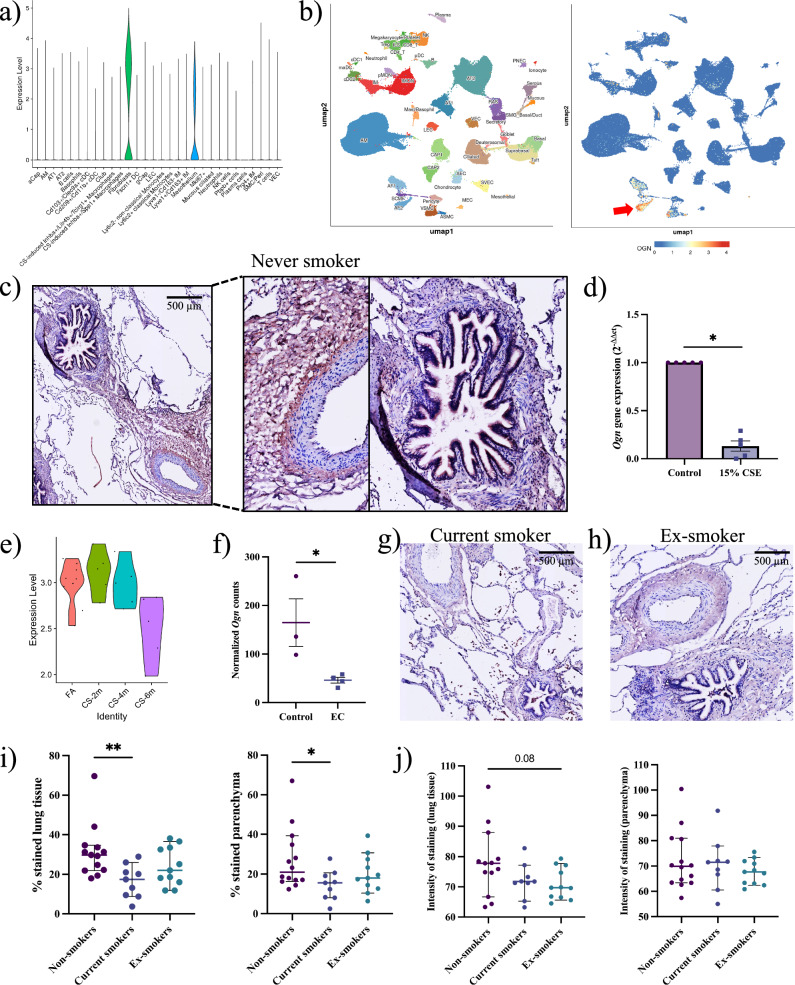
Cigarette smoke reduces fibroblast-derived osteoglycin expression in the lungs. **a** ScRNA-Seq analysis of *Ogn* expression by cell type in murine lungs. **b** ScRNA-Seq analysis showing cell type specificity of *OGN* in the human lung, with red arrow indicating OGN in alveolar fibroblasts. **c** Image of OGN staining in lung tissue of never-smoker (scale = 500 µm), with close-up showing an artery and an airway. **d** *Ogn* gene expression in PCLS treated with 15% CSE (mean ± SEM, N = 5, paired t-test on log-transformed ΔCt values). **e** Pseudobulk ScRNA-Seq of *Ogn* in fibroblasts from lung tissue of mice exposed to two, four, and six months of CS compared to filtered air (FA). **f** Normalized counts from DESeq2 analysis of *Ogn* in fibroblasts upon treatment with a COPD-related exacerbation cocktail (mean ± SEM, N = 3-4). **g**, **h** OGN staining images in lung tissue of current smoker and ex-smoker donor (scale = 500 µm). **i** Percentage (%) of positively stained area for OGN in whole lung tissue and parenchyma in never, current, and ex-smokers. **j** Intensity of positive staining for OGN in whole lung tissue and parenchyma of never, current, and ex-smokers. Statistically significant comparisons: *p < 0.05, **p < 0.01.

To assess cell type-specific expression of OGN in the lungs, we performed single-cell RNA sequencing (scRNA-Seq) analysis of naive murine lung tissue, which showed predominant OGN expression in fibroblasts and, to a lesser extent, in mesothelial cells (Fig. 3a). Confirming these results in human lung tissue, analysis of a public scRNA-Seq dataset (https://lungmap.nl/) showed that 59.06% of alveolar fibroblasts (type 2) express OGN (Fig. 3b, Fig. S3a). Furthermore, OGN gene expression was observed in both MRC5 cells and in primary human lung fibroblasts (Fig. S3b). These data suggest alveolar fibroblasts as the primary endogenous source of OGN in the lungs. In human lung tissue from never-smoker controls, OGN staining was most pronounced around larger arteries, interlobular septa, and pleura, correlating with areas rich in high collagen. (Fig. 3c, Fig. S4a). This suggests that fibroblasts, known to secrete important components of the extracellular matrix, such as collagens, are a likely endogenous source for OGN.

We then investigated the impact of CS on *Ogn* expression by exposing murine PCLS to 15% CSE for 24 h and analyzed *Ogn* expression. CSE treatment significantly reduced *Ogn* expression in PCLS (Fig. 3d). Additionally, in vivo mice exposed to CS for two, four, and six months^26^ showed progressively diminished *Ogn* expression in lung tissue, as revealed by scRNA-Seq analysis (Fig. 3e). Furthermore, *Ogn* was significantly downregulated in murine fibroblasts from resorted organoids treated for 72 h with an exacerbation cocktail (EC) containing COPD-related cytokines (300 pg/mL IL-1β, 300 pg/mL IL-6, 20 ng/mL keratinocyte chemoattractant, and 200 pg/mL TNFα) (Fig. 3f)^24^.

To study the impact of smoking on OGN expression, we compared OGN protein expression in lung tissue of never, current, and ex-smokers (Fig. 3g, h, Supplementary Data 5A). OGN was more abundant in never-smoker lung tissue compared to current and ex-smoker tissue (Fig. 3g, h). Image analysis showed a lower percentage area of OGN-positive staining in current smokers compared to never smokers (Fig. 3i). Interestingly, OGN expression in ex-smokers was not different from current smokers or never smokers, with mean expression values intermediate between these groups, implying a persistent impact of cigarette smoke on OGN expression (Fig. 3i). OGN staining intensity showed no significant differences between never, current, and ex-smokers (Fig. 3j). Next, we assessed whether OGN expression changed in patients with moderate-severe COPD (II/III) or severe-early onset (SEO-)COPD compared to ex-smoker control lung tissue (Fig. S4b, c). In Supplementary Data 5B, the clinical parameters of donors included in this analysis are summarized. In patients with COPD II/III, no significant differences were observed in the percentage area and the average intensity of the OGN staining in both whole lung and parenchyma (Fig. S4d, e). Interestingly, we did observe a tendency for a lower percentage area of OGN staining and higher average staining intensity in the parenchyma of SEO-COPD compared to the control (p = 0.09), which could potentially be explained by strong staining in a specific cell type or region, or active repair responses in surviving cells (Fig. S4f, g). This suggests that while the overall area of OGN-positive tissue may be reduced, the remaining cells within these regions exhibit stronger OGN expression, potentially reflecting an active repair response in surviving cells^27^. Taken together, these data suggest that OGN expression is mainly affected by current smoking.

### Osteoglycin promotes alveolar epithelial growth

**Fig. 4:**
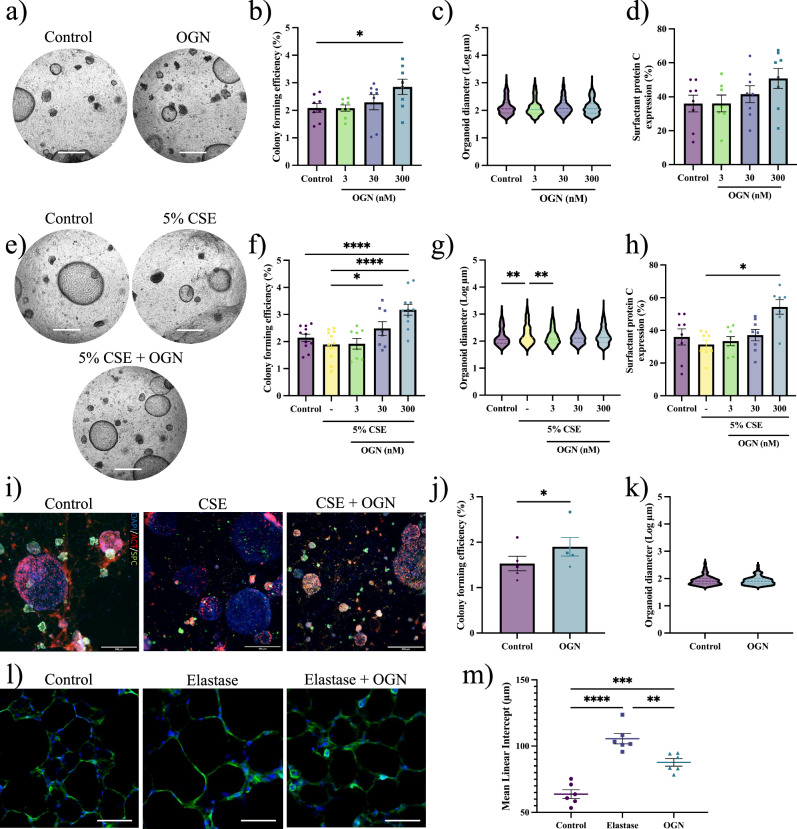
Osteoglycin supports alveolar organoid formation. **a** Brightfield images of murine lung organoids with OGN (300 nM) (scale = 500 µm). **b** CFE of murine organoids (mean ± SEM, N = 8, paired one-way ANOVA, Dunnett test). **c** Log of murine organoid diameter (median, N = 8, Kolmogorov–Smirnov test, Bonferroni correction: α = 0.0167). **d** Immunohistochemistry for pro-surfactant protein C in murine organoids on day 14 (mean ± SEM, N = 6, paired one-way ANOVA, Dunnett test). **e** Brightfield images of CSE-exposed murine lung organoids with OGN (300 nM) (scale = 500 µm). **f** CFE of CSE-exposed murine organoids (mean ± SEM, N = 8–11, paired one-way ANOVA, Dunnett test). **g** Log of CSE-exposed murine organoid diameter (median, N = 8–11, Kolmogorov-Smirnov test, Bonferroni correction: α = 0.0125). **h** Immunohistochemistry for SPC in CSE-exposed murine organoids (mean ± SEM, N = 7–8, paired one-way ANOVA, Dunnett test). **i** Immunofluorescence images of organoids for airway-type (ACT, red), alveolar-type (SPC, green), and DAPI (blue) (scale = 500 µm). OGN is 300 nM. **j** CFE of human organoids with 300 nM OGN (mean ± SEM, N = 5, paired Student T-test). **k** Log of human organoid diameter with 300 nM OGN (median, N = 5, Kolmogorov–Smirnov test). **l** PCLS stained for F-actin (green) and DAPI (blue) (scale = 50 µm) after elastase and OGN (300 nM). **m** LMI measurements following treatments (mean ± SEM, N = 6, one-way ANOVA, Dunnett test). Significant comparisons: *p < 0.05, **p < 0.01, ***p < 0.001, ****p < 0.0001.

OGN, also known as mimecan, is an endogenous small leucine-rich proteoglycan (SLRP) with diverse roles in human biology, including tissue development and fibrosis regulation^28^. We investigated the effects of OGN on alveolar epithelial progenitor cell activation using murine lung organoids. Human recombinant OGN protein provided concentration-dependent growth for lung organoids (Fig. 4a, b) without altering organoid size (Fig. 4c). Interestingly, the proportion of SPC^+^ or ACT^+^ organoids remained unchanged after treatment with OGN (Fig. 4d and Fig. S5a).

In addition, we exposed the organoid cultures to 5% CSE. OGN maintained its regenerative effects without affecting the organoid size and increased the proportion of SPC^+^ organoids in a concentration-dependent manner (Fig. 4e–i). As illustrated in Fig. S5b, CSE treatment alone increased the proportion of airway-type (ACT^+^) organoids. In contrast, additional OGN treatment increased the percentage of alveolar (SPC^+^) organoids, which resulted in a decreased percentage of airway-type (ACT^+^) organoids (Fig. 4i and Fig. S5b). In a two-way ANOVA, there was no significant interaction between OGN concentration and CSE treatment, indicating that OGN’s influence on SPC-expression was despite CSE treatment. Significantly, in a real disease model, using human COPD IV organoids, OGN treatment increased the CFE number without affecting diameter (Fig. 4j, k).

To confirm the effects of OGN on alveolar epithelial growth, we used a mouse PCLS experiment in which we induced emphysematous changes with elastase enzyme^20^. After 16 h incubation with elastase, parenchymal lung tissue damage indicative of emphysema was induced, as evidenced by a significant increase in mean linear intercept (LMI) (Fig. 4l, m). Interestingly, treatment with OGN for 40 h, of which the first 16 h were concomitant with elastase exposure, effectively prevented the induction of lung tissue injury (Fig. 4l, m). Taken together, these data demonstrate the profound potential of OGN on the human and mouse alveolar epithelium, positioning it as a promising therapeutic candidate supporting alveolar epithelial repair.

### Osteoglycin enhances epithelial ROS defense mechanisms and increases fibroblast signaling

**Fig. 5:**
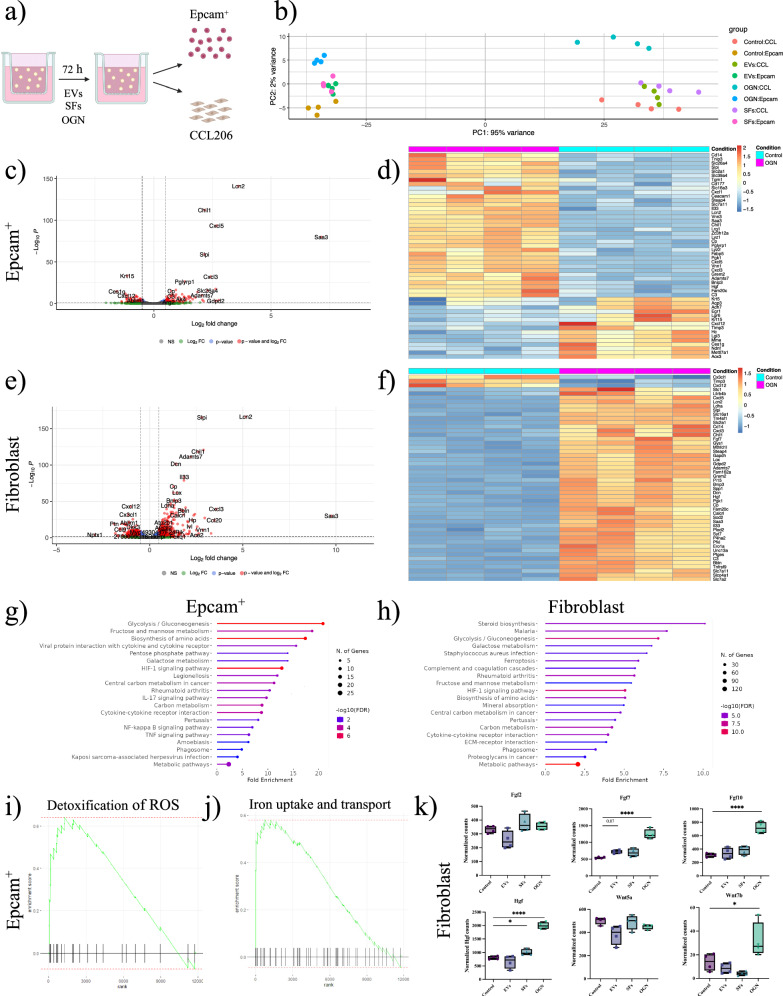
Osteoglycin enhances gene expression for epithelial cell ROS defense mechanisms and increases growth factor expression in fibroblasts. **a** Schematic of organoid RNA sequencing experimental design. **b** PCA plot of epithelial progenitor cells and fibroblasts from murine organoids treated with vehicle, EVs, SFs, or OGN for 72 h (N = 4). **c** Volcano plot illustrating the response to OGN versus control in epithelial progenitor cells (pad j< 0.05 and logFold > 1). **d** Heatmap of top 50 significant genes up- or downregulated in OGN-treated epithelial progenitor cells; shown as z-scores. **e** Volcano plot illustrating the response to OGN versus control in fibroblasts (padj < 0.05 and logFold > 1). **f** Heatmap of top 50 significant genes up- or downregulated in OGN-treated fibroblasts; shown as z-scores. **g** Top 20 upregulated pathways in epithelial progenitors from organoids treated with OGN. **h** Top 20 upregulated pathways in OGN-treated fibroblasts. **i**, **j** Reactome enrichment score for “detoxification of ROS” and “iron uptake and transport” in epithelial progenitor cells. **k** Normalized counts of Fgf2, Fgf7, Fgf10, Hgf, Wnt5a, and Wnt7b in fibroblasts with EVs, SFs, or OGN (mean ± min/max, N = 4). Statistically significant comparisons: *p < 0.05 and ****p < 0.0001.

To investigate the potential mechanism of action, we analyzed transcriptomic changes in murine lung organoids treated for 72 h with EVs, SFs, or OGN. During the experiment, organoids were initially formed by co-culturing epithelial cells and fibroblasts, mimicking the cellular interactions present in the lung microenvironment. Following the 72-h treatment, the organoids were dissociated, and epithelial and fibroblast populations were re-sorted to allow for cell-type-specific transcriptomic analysis using bulk RNA sequencing (Fig. 5a). The 72-h time point was selected as it represents the early stages of organoid formation, capturing the transcriptomic changes associated with the initiation of this process. Principal component analysis (PCA) showed distinct transcriptional profiles for the two cell populations (Fig. 5b). Additionally, OGN-treated Epcam^+^ cells and fibroblasts were transcriptionally different from the other conditions. To confirm cell purity, we compared the proportion of AT2 cells and fibroblasts in all untreated samples based on a cell marker gene signature Supplementary Data 6). The gene signature for AT2 cells was significantly higher in the epithelial cell population, while cells with a fibroblast signature were enriched in the fibroblast population (Fig. S6a).

Paired differential expression analysis using DESeq2 revealed that EV treatment significantly upregulated 67 genes and downregulated 44 genes in epithelial cells and upregulated 1186 genes and downregulated 983 genes in fibroblasts (Fig. S6b–e). SF-treatment had moderate effects, with 27 genes upregulated and 29 downregulated in epithelial cells and 95 genes upregulated and 47 downregulated in fibroblasts (Fig. S6f–i). OGN treatment had more profound effects, with 79 genes downregulated, 118 genes upregulated in epithelial cells, 489 genes downregulated, and 652 upregulated in fibroblasts (Fig. 5c–f). Overall, EVs, SFs, and OGN had the most pronounced transcriptomic effect in fibroblasts compared to epithelial cells.

We then performed GSEA to decipher the observed transcriptomic changes in epithelial cells. Using the KEGG reference database, we identified pathways overrepresented within OGN-modulated genes in Epcam^+^ cells (Fig. 5g) and fibroblasts (Fig. 5h). We also analyzed pathways affected by EVs or SFs (Fig. S7a–c) or OGN (Fig. S7d). OGN treatment upregulated pathways related to Glycolysis/Gluconeogenesis, Biosynthesis of amino acids, HIF-1 signaling, Carbon metabolism, and Metabolic pathways in both cell populations. Reactome analysis showed increased expression of protective pathways, including *Detoxification of reactive oxygen species* and *Iron uptake and transport* in OGN-treated epithelial cells (Fig. 5i, j). Furthermore, OGN treatment enhanced the expression of antiporter subunits *Slc3a2* and *Slc7a11*, facilitating cystine uptake for glutathione synthesis, acting as a co-factor for glutathione peroxidase (Fig. S8a, b). Additionally, OGN increased the expression of superoxide dismutase 2 (*Sod2*), clearing mitochondrial reactive oxygen species (Fig. S8c), while the expression of senescence marker p21 (Cdkn1a) tended to decrease (Fig. S8d). Analysis of AT1 and AT2 markers revealed that the AT1 markers *Emp2*, *Lmo7*, and *Gprc5a* remained unchanged after treatment with EVs, SFs or OGN, and also a composite AT1 gene signature based on the top 10 AT1 marker genes^29^ was unaffected (Fig. S9a). AT2 markers showed a disparate pattern with some (*Sftpb* and *Sftpc*) being unchanged and others (*Sftpa1*) being increased following OGN (but not EVs or SFs) exposure (Fig. S9b). The AT2 signature based on the top 10 AT2 marker genes^29^ was unaffected by OGN (Fig. S9b).

As the transcriptomic changes were most pronounced in fibroblasts, we focused on their expression of growth factors known to be essential mitogens for AT2 cells, including fibroblast growth factor (FGF)2, FGF7, FGF10, HGF, and WNT ligands. We focused on their expression in fibroblasts treated with EVs, SFs, and OGN. OGN notably increased *Fgf7* and *Fgf10* expression in fibroblasts, with a trend towards upregulation with EV treatment (Fig. 5k). *Hgf* expression significantly increased with SFs and OGN treatment. OGN treatment also increased *Wnt7b* expression and showed a tendency towards decreased *Wnt5a* expression (Fig. 5k). Together, these findings suggest enhanced epithelial-fibroblast crosstalk mediated by growth factors upon OGN treatment, potentially contributing to the increased organoid formation.

### An active fragment of osteoglycin supports alveolar epithelial regeneration

**Fig. 6:**
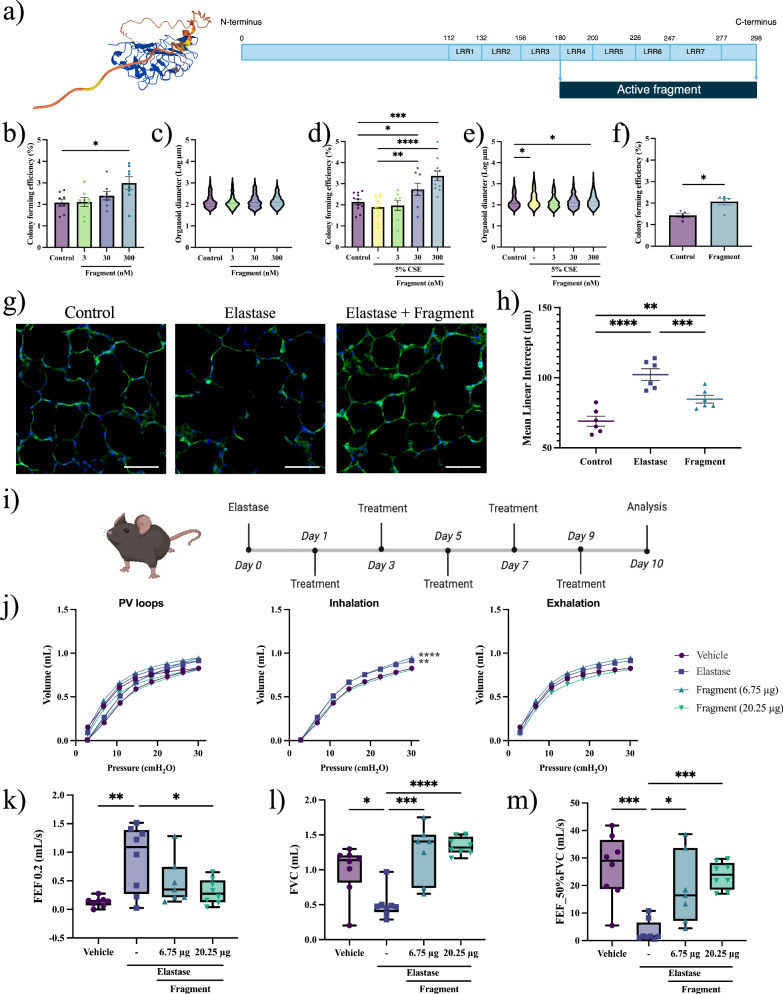
An active fragment of osteoglycin induces alveolar epithelial growth and improves lung function. **a** Predicted alpha-fold structure of osteoglycin, showing the full-length protein comprising seven LRRs and an active fragment (LRR4 - LRR7). **b** CFE of murine organoids treated with OGN fragment (mean ± SEM, N = 8, paired one-way ANOVA, Dunnett test). **c** Log of murine organoid diameter (median, N = 8, Kolmogorov-Smirnov test, Bonferroni correction: α = 0.0167). **d** CFE of CSE-exposed murine organoids (mean ± SEM, N = 8–11, paired one-way ANOVA, Dunnett test). **e** Log of CSE-exposed murine organoid diameter (median, N = 8–11, Kolmogorov-Smirnov test, Bonferroni correction: α = 0.0125). **f** CFE of human organoids (mean ± SEM, N = 5, paired Student T-test). **g** PCLS stained for F-actin filaments (green) and Dapi (blue) (scale = 50 µm) after treatment with vehicle control, elastase, or elastase + fragment (300 nM). **h** Mean linear intercept measurements following treatments (µm, mean ± SEM, N = 6, paired one-way ANOVA, Dunnett test). **i** Schematic of in vivo murine elastase-induced lung injury model. **j** Pressure-volume loops for lung distensibility (N = 7–8, two-way ANOVA, Dunnett test). **k**–**m** Lung function parameters: FEF 0.2, FVC, and FEF_50%FVC measured with FlexiVent (median ± min/max data point, N = 7–8, one-way ANOVA, Dunnett test). Controls are identical to Fig. 4. Statistically significant comparisons: *p < 0.05, **p < 0.01, ***p < 0.001, and ****p < 0.0001.

The protein structure of OGN consists of seven leucine-rich repeats (LRRs). LRRs four to seven contain positively charged residues and are part of the C-terminal chain, forming a tail-like structure composed of amino acids 180 to 298^28^. The specific structure and charge in this protein fragment suggest a role in biological activity or its signaling (Fig. 6a)^28^. Hence, we tested the effects of this OGN fragment on alveolar epithelial progenitor behavior. Similar to full-length OGN, the fragment, which was obtained commercially, increased the CFE of murine organoids in a concentration-dependent manner while organoid size remained unaltered (Fig. S10a and Fig. 6b, c). Higher concentrations of fragment tended to increase the proportion of alveolar (SPC^+^) organoids (Fig. S10b, c) and reduced double positive (SPC^+^/ACT^+^) organoids (Fig. S10c). In the presence of 5% CSE, the fragment increased the CFE and organoid diameter (Fig. 6d, e); however, no significant interaction between OGN fragment concentration and CSE treatment was observed, indicating that the effects of the fragment are irrespective of CSE exposure. Additionally, the fragment significantly increased the proportion of alveolar (SPC^+^) organoids (Fig. S10d) and reduced the proportion of ACT^+^ and double-positive organoids (Fig. S10d), which was irrespective of CSE treatment. Our next aim was to validate the effect of OGN and its active fragment on the growth of AT2 cells within the organoid model. To achieve this, we enriched the initial cell population for AT2 cells by selecting for major histocompatibility complex class II (MHCII^+^) cells and then compared the CFE of the MHCII^+^ cells to the broader Epcam^+^ population (Fig. S11a)^24^. Treatment with OGN or its active fragment resulted in a significant increase in the number of organoids formed in the MHCII^+^ population, while a trend towards increased organoid formation was observed in the Epcam^+^ cell population (p = 0.07) (Fig. S11b). Notably, in MHCII^+^ cultures, treatment with the OGN fragment led to a significant increase in the formation of larger-diameter organoids (Fig. S11c). Additionally, in human organoid cultures derived from COPD IV donors, the CFE of organoids was significantly increased upon treatment with OGN fragment (Fig. 6f). In summary, these results indicate that a smaller active fragment of OGN is sufficient to support alveolar epithelial progenitor behavior.

We then investigated the effect of OGN fragment on differentiated epithelial cells across various lung injury models. Murine PCLS exposed to elastase and treated with active fragment showed significantly decreased LMI, indicating reduced lung injury (Fig. 6g, h). Next, we evaluated whether the active fragment of OGN could improve lung function in vivo in a lung injury murine model. Upon inducing lung injury with elastase, mice received low (6.75 µg) or high (20.25 µg) doses of OGN fragment (Fig. 6i). Elastase treatment induced a decrease in elastic recoil, as shown by a decreased tissue elastance and increased compliance (Fig. S12a–c). Although the active fragment of OGN did not elicit significant changes in elastance and compliance, as compared to elastase, we observed a dose-dependent trend indicating improvement in these lung tissue characteristics (Fig. S12a, c). Elastase did not induce any other significant changes in tissue characteristics, such as resistance, tissue damping, and Newtonian resistance (Fig. S12d–f). Additionally, pressure-volume loops of emphysematous mice displayed a typical significant upward shift (Fig. 6j, k), suggesting that elastase diminished the distensibility of the lungs. Diminished distensibility was absent in emphysematous mice treated with OGN fragment. In addition to lung tissue characteristics, we assessed lung function parameters in different experimental groups. The forced expiratory flow at 0.2 seconds (FEF0.2) significantly increased upon elastase exposure, while the forced vital capacity (FVC) and forced expiratory flow at 50% of forced vital capacity (FEF_50%FVC) were significantly decreased (Fig. 6k–m). Treatment with OGN fragment inhibited the harmful effects of elastase on lung function significantly (Fig. 6k–m). We did not observe any significant changes from the control (vehicle) measurements in forced expiratory volume at 0.2 s, forced expiratory volume at peak expiratory flow, peak expiratory flow, and time to reach peak expiratory pressure (Fig. S12g–j). Furthermore, neutrophil infiltration analysis based on H&E staining in lung tissue showed no significant differences between the experimental groups (Fig. S13). Taken together, the active fragment of OGN ameliorates elastase-induced lung injury in PCLS and improves lung function in mice, thereby underscoring its potential as a promising therapeutic approach.

## Discussion

As there are currently no clinically approved pharmacological treatments to prevent the progression of or reverse tissue destruction in the distal lung, we aimed to discover a new potential drug for (re-)activating alveolar epithelial progenitor cells, leveraging the lung fibroblast secretome. Utilizing a proteomics-guided drug discovery strategy, we identified twelve potential factors, with OGN showing the most pronounced effects on alveolar organoid formation. RNA sequencing demonstrated that OGN is related to pathways involving ROS defense mechanisms and enhanced epithelial-fibroblast crosstalk. OGN expression was reduced in the lung tissue of smoke-exposed mice and current smokers, with a similar tendency in COPD patients. Moreover, an active fragment of OGN, containing LRRs 4–7, showed comparable effects on epithelial progenitor activation as the entire OGN protein and improved lung function in a mouse model of elastase-induced lung injury. These findings identify the active fragment of OGN as a promising drug candidate to prevent the progression of or reverse epithelial tissue destruction in the distal lung.

In the lungs, alveolar epithelial repair is predominantly regulated by AT2 cells, which are instructed by meticulously orchestrated molecular cues from neighboring cells within the alveolar niche^2,15^. The supportive effect of alveolar fibroblasts on epithelial progenitor cells was evident in our organoid assay, where lower organoid numbers were observed when co-cultured with reduced fibroblast numbers. Furthermore, our findings indicate that lung fibroblasts support alveolar organoid formation in a paracrine fashion through the secretion of EVs and SFs. Consistent with these findings, several studies have shown EVs enhance epithelial organoid formation across tissues^30–32^, and that fibroblast-derived SFs are essential for organoid formation^15,33,34^.

Among the candidate factors identified, OGN emerged as the most promising. In the organoid model, an active fragment of OGN (LRRs 4–7) induced alveolar organoid formation, suggesting that this smaller fragment is sufficient for activating alveolar epithelial progenitor cells. Interestingly, this effect was specific to alveolar organoid formation, as the proportion of airway organoids remained unchanged. This specificity could potentially be explained by alveolar epithelial progenitor cells being more susceptible to factors secreted by alveolar fibroblasts, as different progenitor populations may express distinct receptors on their membrane surfaces. This fragment also effectively alleviated elastase-induced lung injury in PCLS and a mouse model. To our knowledge, OGN or its functional fragment has not been studied previously in the context of COPD treatment. While Lee and co-workers revealed that intraperitoneal therapy of mice with OGN improved their whole-body glucose metabolism and insulin homeostasis^35^, most studies on OGN focus on cancer research. To this extent, models of OGN overexpression are usually exploited, which show contradicting effects on epithelial cell proliferation^36–38^. In our organoid model, no changes in organoid size were observed with OGN or its active fragment, implying no discernible changes in proliferation, which could be context-dependent.

By generating transcriptomic signatures of epithelial progenitor cells, we uncovered the upregulation of several protective pathways, including the detoxification of ROS and iron uptake and transport. We have previously shown that anti-oxidants, such as n-acetyl cysteine and mitoquinone, could reverse the negative impact of diesel exhaust particles on lung epithelial progenitors^39^. Additionally, other anti-oxidants effectively reduced lung injury, inflammation, and oxidative stress in mice^40^. Interestingly, the relationship between OGN and the detoxification of ROS has not yet been studied^41^. Besides building on the identified protective pathways, the focus on iron uptake and transport might be crucial in explaining the protective effects of OGN on epithelial progenitors. Genome-wide association studies have highlighted a pathogenetic role for abnormal iron homeostasis in COPD^42,43^. Moreover, intracellular accumulation of iron and lipid peroxidation are associated with ferroptosis, a newly identified type of cell death^44^. Recent studies found that increased ferroptosis and disrupted iron homeostasis were linked to COPD^44,45^.

Lung epithelial progenitor cell behavior is known to be tightly coordinated by alveolar fibroblasts through paracrine signaling of several growth factors, including FGFs and HGF^46,47^. We found that *Ffg7*, *Ffg10*, and *Hgf* expression was significantly increased in response to OGN treatment on fibroblasts. These growth factors stimulate epithelial cell proliferation and survival, which could contribute to the OGN-induced alterations in organoid formation^48,49^. Indeed, FGF7, FGF10, and HGF were previously shown to increase lung organoid formation^17,49^ and FGF7 and FGF10 support maturation of lung organoids toward distal lung lineages^50,51^. In addition, FGF signaling was shown to have antifibrotic effects in experimental pulmonary fibrosis through epithelial progenitor proliferation and inhibition of TGF-β^52^. As the receptor with which OGN interacts in the lung is unknown, it remains unclear whether OGN directly influences alveolar epithelial cell behavior or indirectly through the intermediary action of fibroblasts. However, the total number of differentially expressed genes in fibroblasts following OGN treatment is significantly higher compared to those in the epithelial cell population, suggesting a predominant effect on fibroblast function. This suggests that fibroblasts may serve as a mediator through which OGN indirectly promotes organoid formation and epithelial repair. Future studies should aim to identify the receptor(s) involved in OGN signaling and further delineate the direct versus indirect effects of OGN on alveolar epithelial cells. Additionally, while OGN treatment upregulated key pro-regenerative factors such as FGF7, FGF10, and HGF in fibroblasts, the effects of EVs and SFs on these factors appeared somewhat distinct. This difference suggests that the observed regenerative effects may not be solely linked to OGN, indicating that EVs and SFs could exert independent effects mediated through other messenger molecules.

In this study, we describe for the first time the distribution of OGN in human lung tissue on both a transcriptomic and protein level. We observed proportionally lower OGN protein expression in the lung tissue of current smokers compared to non-smokers, with ex-smokers showing intermediate levels. This suggests a lasting effect of cigarette smoke exposure on OGN expression. These findings align with Lin and colleagues, who reported reduced OGN mRNA expression in lung tissue of severe COPD patients^53^. Interestingly, Huang and colleagues reported upregulation of OGN in lungs and myofibroblasts obtained from lung fibrosis murine models^54^. Koloko Ngassie et al. demonstrated a positive correlation between increasing age and enhanced OGN protein expression in human lung tissue derived from patients with no history of chronic lung disease^55^. Considering our data, these findings suggest that OGN expression in the healthy human lung increases with age, whereas this process appears compromised in individuals who smoke. Such impairment could contribute to the hampered alveolar repair and regeneration mechanisms observed in COPD patients. Since this effect is also observed in healthy smokers, it is plausible that impaired OGN expression represents an early event in the path to COPD progression or that a secondary insult is required to drive disease development.

Based on our various disease models, we speculate that therapeutically supplementing COPD patients with OGN or its active fragment could potentially (re-)activate lung epithelial repair or slow down the progression of tissue damage. Our study predominantly utilized lung tissue and organoids derived from severe COPD patients. While these models effectively demonstrated OGN’s potential to restore epithelial progenitor cell function even in highly impaired epithelial populations, earlier therapeutic intervention may have even greater benefits. At earlier stages of COPD, the pool of alveolar epithelial progenitors is likely larger and more responsive to therapeutic cues, making interventions more effective in promoting repair and regeneration. Moreover, slowing disease progression at these stages could preserve lung function and prevent significant tissue damage. These considerations highlight the importance of further research to determine the therapeutic potential of OGN in less severe COPD models and effectiveness across different stages of disease progression. While alveolar epithelial repair is a crucial step toward regaining functioning alveolar tissue, it is essential to consider that the complete restoration of alveolar function involves addressing the entire alveolar niche, which includes various cell types and extracellular matrix components. To further this research, it is crucial to determine which COPD patients would benefit most from this therapy, at what stage of the disease, and to consider the differences between current and former smokers. Additionally, it is worth noting that OGN is expressed in various tissues, including bone, muscle, and adipose tissue^28^, and its glycosylation and splice variants may confer cell-type and tissue-specific effects, necessitating further studies to fully understand its mechanism of action.

When contemplating the therapeutic application of OGN as a regenerative agent for (re-)activating alveolar epithelial repair, careful consideration of potential adverse effects associated with exogenous administration of this protein is essential. As a proteoglycan, OGN has been linked to the induction of fibrosis^56^. For instance, Shi et al. demonstrated that microRNA-140 overexpression inhibits pulmonary fibrosis by downregulating OGN through the Wnt signaling pathway^57^. The role of OGN in fibrosis regulation extends beyond the lungs, encompassing organs like the heart and kidney^28^. However, our RNAseq analysis offers a contrasting perspective, indicating a tendency for downregulation of the predicted gene signature for myofibroblasts and fibrosis-related genetic markers upon OGN treatment. It is hypothesized that OGN regulates fibrosis through the crosslinking of collagen via the N-terminus^56,58^. Following this rationale, therapeutic use of OGN might induce fibrosis by increasing cross-linking. However, in our assays, we show that a fragment encompassing LLRs four through seven exhibits similar effects on epithelial progenitor cell behavior. This may present an attractive approach to circumvent fibrosis initiation in individuals by eliminating the structural component crosslinking activities of the entire OGN protein.

A limitation of this study is the relatively small number of patients included in the different analyses. The variability observed in the data across these studies could be attributed to the heterogeneity of COPD, variations in patient inclusion criteria, and differences in analysis methods. In the context of IHC staining on OGN expression in the lungs, we must acknowledge that our examination is confined to the remaining lung tissue. Particularly in end-stage COPD patients, substantial tissue damage exists, posing challenges to the accurate quantification of OGN expression across different disease stages. Another potential limitation is the use of human lung fibroblast-derived EVs and SFs in a murine organoid system. While this decision was made to align with the ultimate goal of developing a therapy for human use, it introduces the possibility that certain species-specific effects may be lost. To address this limitation, we have also cultured organoids derived from human lung tissue to provide complementary insights.

In conclusion, lung fibroblasts support the formation of organoids through the secretion of EVs and SFs. Using a proteomics-guided drug discovery strategy, we identified OGN to activate alveolar epithelial progenitor cells. OGN induces a wide range of transcriptional changes, including the upregulation of protective pathways in epithelial progenitors and growth factor-mediated epithelial-fibroblast crosstalk. Expression of OGN was reduced in response to cigarette smoke and the lung tissue of smokers. Furthermore, an active fragment of OGN was shown to have similar properties on epithelial progenitor activation and improved lung function parameters in mice with lung injury. While this study focused on COPD, potential benefits of OGN could extend to other conditions marked by deficient epithelial progenitor function across organs.

## Methods

### Antibodies and reagents

An anti-pro-SPC antibody (AB3786) was purchased at Sigma Aldrich, and acetylated α tubulin (sc-23950) was obtained from Santa Cruz. Secondary antibodies for Alexa Fluor™ 488 (A21206) and 568 (A10037) were purchased at Thermo Fisher. Recombinant human OGN full protein was purchased at Biorbyt (orb383003), and the human OGN fragment (amino acid 180-298) was obtained from LifeSpan Biosciences (LS-G15022). Pancreatic porcine elastase for in vitro (E0258-5MG) and in vivo (324682-250U) experiments were obtained from Sigma Aldrich.

### Cell culture

Mouse fibroblasts, CCL206 cells (ATCC, Mlg2908), were cultured in DMEM/F12 medium (Gibco) supplemented with 10% (v/v) fetal bovine serum (FBS) (Sigma Aldrich, 12103 C), 100 U/mL penicillin/streptomycin (Gibco, 15070-063), 2 mM L-glutamine (Gibco, 25030-024), and 1% amphotericin B (Gibco, 15290026) within a humidified atmosphere under 5% CO_2_/95% air at 37 °C. The human fetal mesenchymal lung fibroblast MRC5 cell line (Sigma Aldrich, 05081101) was cultured in Ham’s F12 medium (Thermo Fisher, 11320033) supplemented with 10% (v/v) FBS, 100 U/mL penicillin/streptomycin, and 2 mM L-glutamine within a humidified atmosphere under 5% CO_2_/95% air at 37 °C.

For organoid experiments, before both fibroblast types (CCL206 and MRC5) were co-cultured with primary epithelial cells, the proliferation of fibroblasts was inactivated by incubation with mitomycin C (10 µg/mL) in growth medium (Sigma Aldrich, M4287-5X2MG) for 2 h. After incubation with mitomycin C, the fibroblasts were washed with warm PBS and then recovered for 1 h in mitomycin C-free growth medium.

### Purification of EVs and SFs using ultrafiltration and size exclusion chromatography

MRC5 lung fibroblasts were initially expanded from a single T75 flask to 13 T175 flasks through several passaging steps. Once the MRC5 cells reached approximately 80% confluency, the cells were serum starved for a period of 72 h. The conditioned medium was collected and centrifuged at 4000 *g* for 15 min, before filtering using a 0.45 µm bottle top filter. The conditioned medium was then concentrated using 100 kDa Amicon Ultra-15 Centrifugal Filter Units (Merck, UFC900324) by repeated centrifugation at 4000 *g* at 4 °C to a final volume of ~500 µL. A 30-cm-long, 1-cm-diameter column packed with Sepharose CL-2B (Cytiva, 17-0140-01) was washed with PBS prior to loading the concentrated medium. Thereafter, 24 × 1 mL fractions were collected from the column. Size exclusion column (SEC) fractions 8 till 12 were considered to be the EV-enriched fractions, while fractions 18 till 22 served as the SF-enriched fractions. Both the EV- and SF-fractions were pooled and concentrated using 100 kDa Amicon Ultra-4 Centrifugal Filter Units (Merck, UFC801024) to a final volume of ~600 µL. Subsequently, the EV- and SF-enriched samples were sterilized using Costar Spin-X centrifuge tube filters (Merck, CLS8160-96EA) with a 0.22 µm pore size. Samples were stored at −80 °C until further use. For the treatment of organoid cultures, we used concentrations of EVs and SFs that showed the strongest impact on colony forming efficiency in murine organoids (Fig. S2). Multiple purification batches were applied for each organoid experiment to ensure consistency and reproducibility.

### Animals

Lung harvesting from naive C57BL/6 J mice (eight to fourteen weeks old) for organoid experiments and PCLS was performed at the Central Animal Facility (CDP) of the University Medical Center Groningen (UMCG) per the national guidelines and upon approval of the experimental procedures by CDP and the Institutional Animal Care and Use Committee (IACUC) of the University of Groningen, under AVD1050020209205. A total of 66 mice were used, with an equal ratio of male and female animals. For the elastase in vivo study, we used 48 C57BL/6 J mice, with a 1:1 ratio of male to female animals. Mice were anesthetized by subcutaneous injection of 400 mg/kg Ketamidor® (Alfasan) and 1 mg/kg Dexdomitor® (Orion Pharma). Animals were housed conventionally under a 12-h light-dark cycle and received food and water ad libitum.

### Human material

Human lung tissue was obtained from lung transplant donors in strict adherence to the Research Code of the UMCG, as stated on https:// umcgresearch.org/w/research-code-umcg as well as national ethical and professional guidelines, including the Code of Conduct for Health Research and Declaration of Helsinki. The use of left-over lung tissue in this study was not subject to the Medical Research Human Subjects Act in the Netherlands, as confirmed by a statement of the Medical Ethical Committee of the UMCG, and therefore exempt from consent according to national laws (Dutch laws: Medical Treatment Agreement Act (WGBO) art 458 / GDPR art 9/ UAVG art 24). Human lung tissue was acquired from extra tissue left over after lung surgeries, which exceed the amount needed for clinical care purposes. All samples and clinical information were blinded before experiments.

### Primary alveolar epithelial isolation

Isolation of murine primary alveolar epithelial cells, in brief, Epcam^+^ cells (CD31^-^/CD45^-^/CD326^+^), was based on previously published protocols^3^. In brief, the lungs of mice were flushed through the heart with PBS, instilled with dispase (Corning, 354235), and incubated at room temperature for 45 min. To obtain a single-cell suspension, lung tissue was digested with DNase 1 (VWR, A3778.0500). Using the QuadroMACS™ Separator (Miltenyi Biotec, 130-091-051) and a mix of antibody-bound magnetic microbeads, the cell suspension was negatively selected for CD31 (Miltenyi Biotec, 130-097-418) and CD45 (Miltenyi Biotec, 130-052-301). Subsequently, to obtain Epcam^+^ cells, CD31^-^/CD45^-^ cells were positively selected with anti-mouse CD326 microbeads (Miltenyi Biotec, 130-105-958).

Human lung tissue was stored in MACS® Tissue Storage Buffer (Miltenyi Biotech, 130-100-008) until further processing. Lung tissue was cut into small pieces (~1 mm^3^) and transferred to a dissociation mixture containing 1% penicillin/streptomycin, 1 mg/mL collagenase/dispase (Roche, 11097113001), and 1.8 µg/mL DNase 1 in PBS. The tissue was further dissociated using a gentleMACS™ Octo Dissociator with heaters (130-096-427, Miltenyi Biotec) for 20 min at 37 °C. The obtained single-cell suspension was washed, and red blood cells were lysed using lysis buffer (ammonium chloride (155 mM), potassium bicarbonate (1 mM), titriplex III (0.001 mM), and 10 µg/mL DNase 1 in ultra-pure water) for 10 min at 4 °C. Selection for EpCAM^+^ cells was similar to that described above for murine lung tissue. Clinical data corresponding to the human lung tissue samples used in this study are provided in Supplementary Data 7.

### Epithelial organoid culture

For murine organoids, freshly isolated Epcam^+^ cells were combined with CCL206 murine lung fibroblasts at a 1:1 ratio (10,000 cells each) in DMEM/F12 containing 10% (v/v) FBS. The cell suspension was then diluted 1:1.5 (v/v) with Corning® Matrigel® Membrane Matrix (Corning, 356234) and was then seeded into transwell inserts (Greiner, 662641) in 24-well plates (100 µL/insert). Similarly, human organoids were generated by co-culturing freshly isolated EpCAM^+^ cells with proliferation-inactivated MRC5 lung fibroblasts. The Matrigel™ was allowed to solidify at 37 °C for 30 min. Upon solidification, 410 µL of organoid medium (DMEM/Ham’s F12 supplemented with 5% FBS, 1% penicillin/streptomycin, 1% L-glutamine, 1% amphotericin B, 0.025‰ epidermal growth factor (EGF) (Sigma Aldrich, SRP3196-500UG), 1% insulin-transferrin-selenium (Gibco, 51300044), and 1.75‰ bovine pituitary extract (Thermo Fisher, 11568866)) was added underneath the insert. On the day of seeding, 10 µM Y-27632 dihydrochloride (Axon, 1683) was added to inhibit Rho-Kinase selectively. Organoid cultures were cultured at 37 °C with 5% CO_2_. The medium was refreshed every 2–3 days. The total number and diameters of organoid structures (>50 µm) was measured on day 14 using NIS-Elements with bright field microscopy (20x magnification). For the proteomics-guided drug discovery strategy, preliminary screening was conducted with N = 3 organoids, and additional experiments (N = 2) were performed for compounds showing promising results to confirm their effects.

### Cigarette smoke extract

To generate 100% CSE, the smoke from two 3R4F research cigarettes (Tobacco Research Institute) without a filter was introduced into 25 mL of warm fibroblast culture medium^3^. The smoke was delivered into the medium using a peristaltic pump (Watson Marlow 323 E/D) at a speed of 45 rpm. CSE was freshly prepared before each set of experiments. For organoid experiments, we used 5% CSE in organoid growth medium.

### Immunofluorescence staining organoids

Organoids were fixed in a 1:1 (v/v) mixture of acetone and methanol for 12 min at −20 °C^3^. After fixation, 1 mL of PBS with 0.02% sodium azide (Merck, 6688) was added to the well underneath the insert. Organoids were kept at 4 °C for 1 week after fixation. Organoids were blocked using a blocking solution containing 5% bovine serum albumin (BSA), 2% normal donkey serum (Jackson Immuno Research, 017-000-121), and 0.1% Triton-X100 in PBS overnight at 4 °C. The next day, surfactant protein C (SPC) and acetylated α tubulin (ACT) primary antibodies diluted 1:200 in 2% BSA, 2% normal donkey serum, and 0.1% Triton-X100 in PBS were added for 48 h. Organoids were then washed three times with PBS for 30 min, and secondary antibodies Alexa Fluor™ 488 and 568 diluted 1:200 in 2% BSA, 2% normal donkey serum, and 0.1% Triton-X100 in PBS were added for 2 h at room temperature. After washing three times with PBS for 30 min, the organoids on the insert membrane were transferred to a glass slide with a mounting medium containing DAPI, and a coverslip was applied afterward. The slides were kept at 4 °C, and confocal images were acquired using a Nikon Eclipse Ti2 microscope.

### Organoid resorting to regain fibroblasts and epithelial cells

For organoid resorting, a mixture of 300,000 Epcam^+^ cells and 300.000 CCL206 fibroblasts was seeded in a 1 mL solution of Matrigel diluted 1:1.5 (v/v) with DMEM/F12 (supplemented with 10% FBS) and added to one well of a 6-well plate. After the Matrigel solidified for an hour, 2 mL of organoid culture medium was added on top of the Matrigel, including EVs (10^9^ EVs/mL), SFs (30 µg/mL), or OGN (10 µg/mL). After three days, dispase (Corning, 354235) was added to each well for 30 min at 37 °C to dissociate the Matrigel. MACS buffer (MACS rinsing solution (Milteny Biotec, 130-091-222) premixed with BSA (Milteny Biotech, 130-091-376)) was added to stop the dispase activity. Organoids were collected and centrifuged at 300 *g* for 5 min. The pellets were resuspended in 5 mL diluted trypsin (1:5 in PBS, v/v) (T7409, Sigma-Aldrich) for 5 min at 37 °C, after which 9 mL DMEM/F-12 supplemented with 10% FBS was added to neutralize trypsin action. The cell pellets were incubated with CD326 microbeads for 20 min and resuspended in MACS buffer. The cell suspensions were introduced to the QuadroMACS™ Separator system to obtain CD326^-^ fibroblasts and CD326^+^ (Epcam^+^) epithelial cells derived from organoids, which were then used for further experimentation.

### Bulk RNA sequencing analysis

Total RNA was extracted from cells resorted from organoids using the NucleoSpin RNA isolation kit (Bioké, 740955.50) according to the manufacturer’s instructions. Bulk RNA sequencing (RNAseq) on resorted Epcam^+^ and fibroblasts from organoids was performed by GenomeScan (www.genomescan.nl) using an Illumina NovaSeq 6000 sequencer. The analysis procedure comprised several steps, including data quality control, adapter trimming, alignment of short reads, and feature counting. To ensure the integrity of the library preparation, calculations were performed to assess the ribosomal (and globin) content. Additionally, checks were conducted to identify potential samples and barcode contaminations. Quality control tools, such as FastQC v0.34 and FastQA, were employed to establish a set of standard quality metrics for the raw dataset. Prior to alignment, Trimmomatic v0.30 was utilized to remove adapter sequences from the reads. The reads of each sample were aligned against the ensemble mouse reference GRCm38 (patch 6). Principal component analyses were performed in R using the R package DESeq2 in order to visualize the overall effects of experimental covariates as well as batch effects. The same R packages were used to identify differentially expressed genes (DEGs) between control and treated samples following standard normalization procedures. Gene set enrichment analysis (GSEA) of the top 50 differentially regulated genes was performed with ShinyGO 0.77 (www.bioinformatics.sdstate.edu/go/). Kyoto Encyclopedia of Genes and Genomes (KEGG) was used as reference database, and the statistically significant pathway enrichment with FDR q value < 0.05 are reported.

### Proteomic analysis

Proteomic data from MRC5-derived EVs and SFs were obtained from 4 biological replicates. Liquid chromatography-tandem mass spectrometry (LC-MS) was employed for unbiased shotgun proteomic analysis. In-gel digestion was performed as described previously^59^ using 1 µg total protein in the EV-enriched samples and 75 µg total protein for the SF-enriched samples. LC-MS-based proteomics analyses were performed as described previously^60^ using 0.5 µg digested total protein starting material from the EVs or 1 µg digested total protein starting material from the SFs. LC-MS raw data were processed with Spectronaut (version 15.4.210913, Biognosys) using the standard settings of the directDIA workflow with a human SwissProt database (www.uniprot.org, 20350 entries). For the quantification, local normalization was applied, and the Q-value filtering was adjusted to the classical setting without imputing.

### Immunohistochemical staining on human lung slides

Control and COPD human lung tissue was obtained from leftover material at the UMCG and St. Mary’s Hospital, Mayo Clinic Rochester, MN. This staining was part of the HOLLAND (HistopathOlogy of Lung Aging aNd COPD) cohort^55^. Immunohistochemical (IHC) staining on human lung slides was performed as described previously^55^. Lung tissue was embedded in paraffin and cut into 6 µm thick sections. These sections were deparaffinized and rehydrated, after which antigen retrieval was performed with 10 mM citrate buffer (pH 6). Endogenous peroxidase activity was blocked by 0.3% hydrogen peroxidase (H_2_O_2_), followed by overnight incubation at 4 °C with a primary OGN antibody (1:400) (Abcam, ab168348) in 1% BSA-PBS. Subsequently, sections were washed and incubated with horseradish peroxidase (HRP)-conjugated secondary antibody diluted 1:100 in 1% BSA-PBS containing 2% normal human serum. Staining was then visualized upon 5 min incubation with Vector NovaRED Substrate (VectorLaboratories, SK-4800). Sections were then counterstained with hematoxylin, mounted, and scanned using a Hamamatsu NanoZoomer 2.0HT digital slide scanner at 40x magnification.

### OGN expression analysis in human lung slides

Analysis of the intensity and area of positive OGN staining in whole lung tissue and parenchyma were analyzed as described previously^55^. Briefly, Aperio ImageScope software V.12.4.3 (Leica Biosystems) was used to extract images from the scans. For whole lung tissue analysis, scanned images were used after removal of artefacts. This step was followed by extracting specific areas, including airway wall, bronchial epithelium, and blood vessels, using Adobe Photoshop software (Adobe Inc. CA), to analyze OGN expression in the parenchyma. Fiji/ImageJ software was used to quantify the intensity and area of positive staining of OGN in whole tissue and parenchyma. The formula used to calculate the percentage of area stained positive for the protein is as follows:

1 $${Area}\left( \% \right)=\frac{{Area}({NovaRed})}{{Area}({Total})}\times 100 \%$$

For the quantification of the intensity of the staining, pixel intensities, ranging from 0 to 255 of separated NovaRed images were analyzed with Fiji/ImageJ. A value of 0 corresponds to the darkest shade, while 255 represents the lightest shade. This reciprocal intensity is directly proportional to the quantity of positive NovaRed pixels detected in the image analysis and is calculated with the following equation:

2 $${Mean\; intensity}=255-\frac{{Sum\; of\; intensities\; of\; pixels\; positive\; for\; NovaRed}}{{Total\; number\; of\; pixels\; positive\; for\; NovaRed}}$$

### Precision-cut lung slices

PCLS were prepared as described previously^20^. Murine lungs were inflated with 1.5 mL 1.5% (w/v) low melting agarose solution (Gerbu Biotechnik). After inflation, the agarose was allowed to solidify at 4 °C for 15 min before the lungs were harvested. A tissue slicer (Leica VT 1000 S Vibratome line) was used to cut lung slices with a thickness of 250 µm. The lung slices were extensively washed and subsequently cultured in DMEM (Gibco, 42430-025) supplemented with sodium pyruvate (1 mM), MEM non-essential amino acids mixture (1:100, Gibco, 11140-050), gentamycin (45 µg/mL, Merck, G1397), penicillin/streptomycin (100 U/mL), and amphotericin B (1.5 µg/mL, Gibco, 15290-026) in 12-well culture plates, using three slices per well. Slices from the same mouse were matched based on lung region (middle region of the left lung lobe or middle region of superior right lung lobe). To induce emphysematous changes, matched slices were treated with 2.5 µg/mL elastase for 16 h. Slices were treated with 10 µg/mL OGN or 4.48 µg/mL OGN active fragment for 40 h, overlapping with the 16 h elastase treatment.

### Immunofluorescence staining PCLS

PCLS were fixed for 15 min at 4 °C with 4% paraformaldehyde (Sigma Aldrich, P6148) and then washed with PBS. To visualize the parenchyma of PCLS, actin filaments were stained with Alexa Fluor™ 488 Phalloidin (Thermo Fisher, A12379) for 15 min at room temperature. After incubation, the slices were washed with PBS and transferred onto a glass slide with two drops of mounting medium containing DAPI (Abcam, 104139). Fluorescence imaging was performed using a confocal laser scanning microscope equipped with a true confocal scanner (SP8 Leica) using a 20x lens. All images were acquired within the linear range, with an image resolution of 1024 × 1024 pixels and a pinhole size of 1 Airy unit to avoid local saturation. The presented images represent a single z-scan. To assess the degree of lung injury in PCLS, we measured the mean linear intercept (LMI), which represents the average distance of free airspace in 5 fields per animal, as previously described^20^.

### Quantification of the degree of lung injury in PCLS

To assess the degree of lung injury in PCLS, we measured the LMI, which represents the average distance of free airspace, in 5 fields per animal, as previously described^20^. However, due to the ex vivo treatment with elastase, we were unable to follow the standard procedure of fixing the murine lungs at 25 cmH_2_O for 24 h, which is typically used for LMI determination. In this PCLS model, the use of varying pressures to fill the murine lungs with agarose results in variations in LMI measurements between animals. Nevertheless, each animal served as its own control, eliminating the variability caused by different pressure conditions applied to the lungs. However, caution should be exercised when extrapolating these numbers to the in vivo setting.

### Elastase in vivo model

For this study, pulmonary emphysematous changes were induced by intratracheal instillation of pancreatic porcine elastase (40 U/kg body weight) in 40 µL sterile PBS on day zero. Animals were treated every other day from day zero till day nine (5 treatments in total) with OGN fragment (6.75 µg or 20.25 µg). After ten days, mice were sacrificed by exsanguination under anesthesia, after which the therapeutic effects were examined. To ensure objective analysis, samples for in vivo experiments were blinded before analysis.

### Lung function measurements

Respiratory function was measured using a FlexiVent system module 2 (Scireq). Mice were anesthetized with Dexdomitor® and Ketamine® and a muscle relaxant, rocuronium bromide (Fresenius Kabi, 10 mg/mL) was administered. Mice were ventilated with a tidal volume of 10 mL/kg at a frequency of 150 breaths/min in order to reach a mean lung volume similar to that of spontaneous breathing. Lung function parameters were assessed using pre-installed protocols for SnapShot, Primewave perturbation, and forced expired volume maneuver using the Flexiware V8.3.0 software. Three recordings per animal were taken.

### Statistical analysis

Results are shown as mean ± standard error, with sample size and repeats in figure legends. Four normality tests (D’Agostino-Pearson, Anderson-Darling, Shapiro-Wilk, Kolmogorov-Smirnov) were conducted; if three indicated normality, parametric tests were applied. One-Way ANOVA with Dunnett’s multiple comparisons was used for group comparisons, and unpaired/paired two-tailed Student’s *t* test or Mann–Whitney and Wilcoxon tests for two-group comparisons. Significance was set at p < 0.05. Analyses were done in GraphPad Prism 10 and RNA sequencing analysis in R Studio.

## Supplementary information

Supplementary Information

Supplementary Information

Supplementary Information

Supplementary Information

Supplementary Information

Supplementary Information

Supplementary Information

Supplementary Information

## Data Availability

All data associated with this study are in the paper or the Supplementary Materials. The complete RNA sequencingdataset is available in GEO at GSE271272. The proteomics dataset of the secretome of human lung fibroblasts isavailable in the PRIDE proteomics identifications database (PXD053766). For material requests, researchers should contact A.N. or R.G.
