# Supplementary material for: Fibroblast-derived osteoglycin promotes epithelial cell repair
Source: NPJ Regen Med. 2025 Mar 25;10:16. doi: 10.1038/s41536-025-00404-3 (PMC11937367; doi:10.1038/s41536-025-00404-3)
Supplement: Supplementary file 1 — Supplementary Information [file 41536_2025_404_MOESM1_ESM.pdf]

Supplementary Information for:

Fibroblast-derived osteoglycin promotes epithelial cell repair

Luke van der Koog *et al.*

Corresponding author: Reinoud Gosens, r.gosens@rug.nl

The PDF includes:

Figures. S1 to S13

Supplementary Figures:

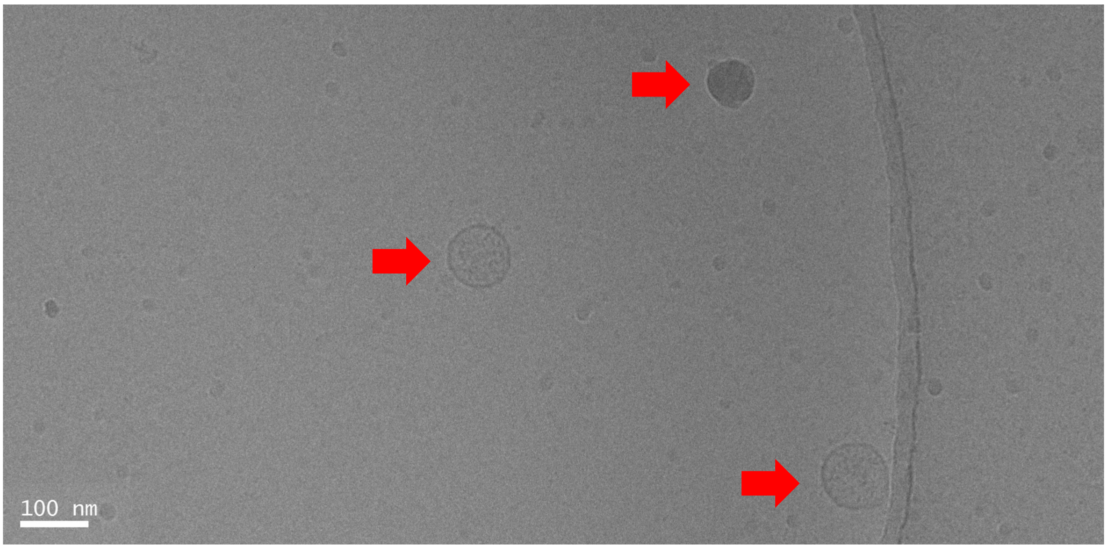

Fig. S1. Lung fibroblast-derived EVs visualized by Cryo-TEM.

Cryo-TEM image of pooled lung fibroblast-derived EV-enriched fractions. Red arrows indicate EV-structures. Scale = 100 nm.

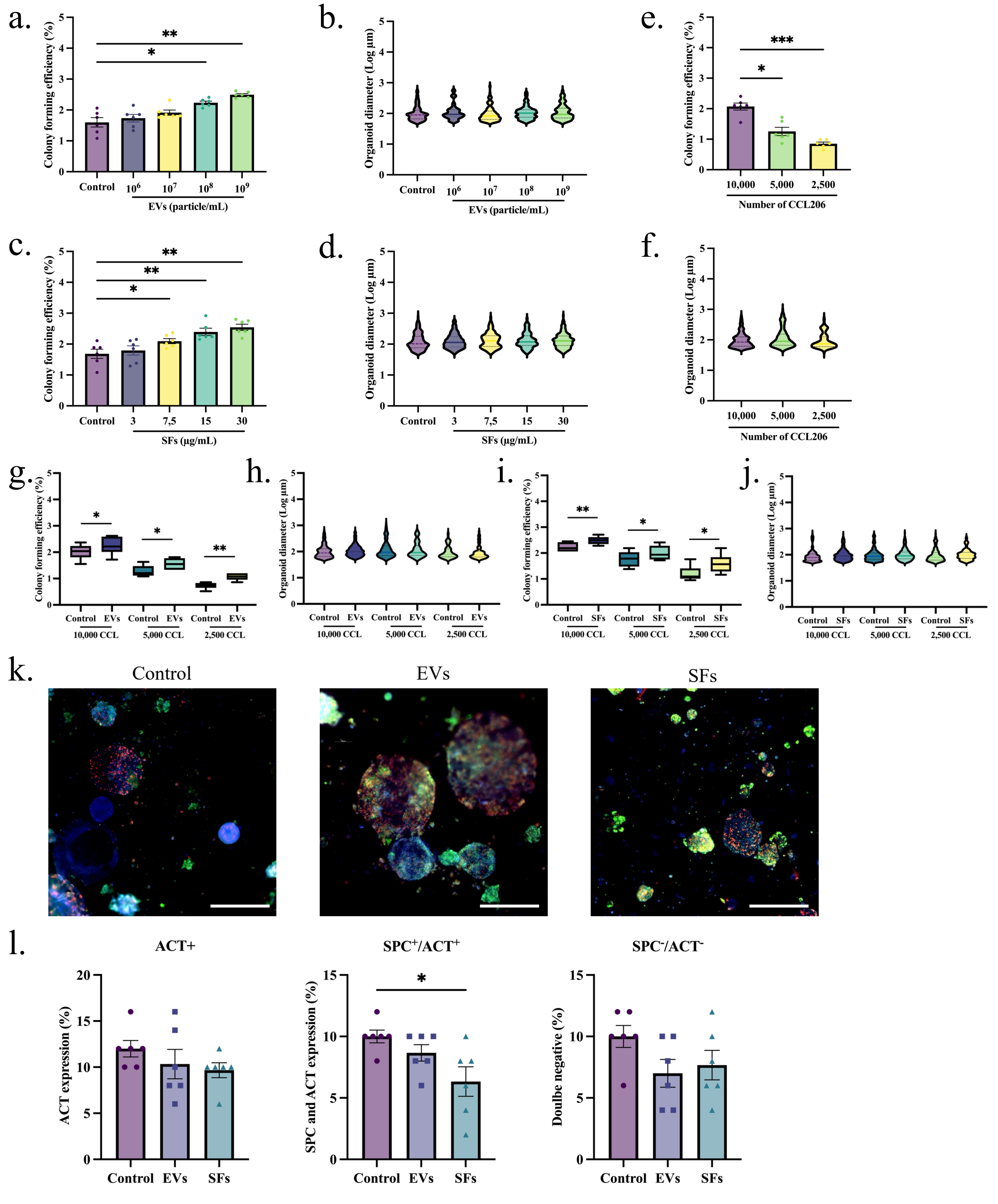

Fig. S2. Lung fibroblast-derived EVs and SFs support murine alveolar organoid formation.

a Colony forming efficiency of murine organoids with increasing concentrations of EVs (mean ± SEM, N=6, paired Friedman test). **b** Log of murine organoid diameter with increasing concentrations of EVs (median is shown, N=6, Kolmogorov-Smirnov test (after Bonferroni correction: α = 0.0125)). **c** Colony forming efficiency of murine organoids with increasing concentrations of SFs (mean ± SEM, N=6, paired

Friedman test). **d** Log of murine organoid diameter with increasing concentrations of SFs (median is shown, N=6, median is shown, N=6, Kolmogorov-Smirnov test (after Bonferroni correction: $\alpha = 0.0125$). **e** Colony forming efficiency of murine organoids with decreasing lung fibroblasts (CCL206) (mean ± SEM, N=6, paired Friedman test). **f** Log of murine organoid diameter with decreasing numbers of lung fibroblasts (CCL206) (median is shown, N=6, Kolmogorov-Smirnov test (after Bonferroni correction: $\alpha = 0.0025$)). **g** Colony forming efficiency of murine organoids with decreasing numbers of lung fibroblasts (CCL) treated with EVs (10^9 EVs/mL) (mean ± SEM, N=6, paired Friedman test). **h** Log of murine organoid diameter with decreasing numbers of lung fibroblasts (CCL) treated with EVs (mean ± SEM, N=6, Friedman). **i** Colony forming efficiency of murine organoids with decreasing numbers of lung fibroblasts (CCL) treated with SFs (30 µg/mL) (mean ± SEM, N=6, paired Friedman test). **j** Log of murine organoid diameter with decreasing numbers of lung fibroblasts (CCL) treated with SFs (30 µg/mL) (mean ± SEM, N=6, Friedman). **k** Representative immunofluorescence images of organoids for airway-type (ACT, red), alveolar-type (SPC, green), and DAPI (blue) (scale = 500 µm). **l** Immunohistochemistry quantification for ACT^+, SPC^+/ACT^+, and SPC^-/ACT^- organoids (mean ± SEM, N=6, paired one-way ANOVA with Tukey test for multiple testing). Statistically significant comparisons are represented by *p < 0.05, **p < 0.01, and ***p < 0.001.

a.

b.

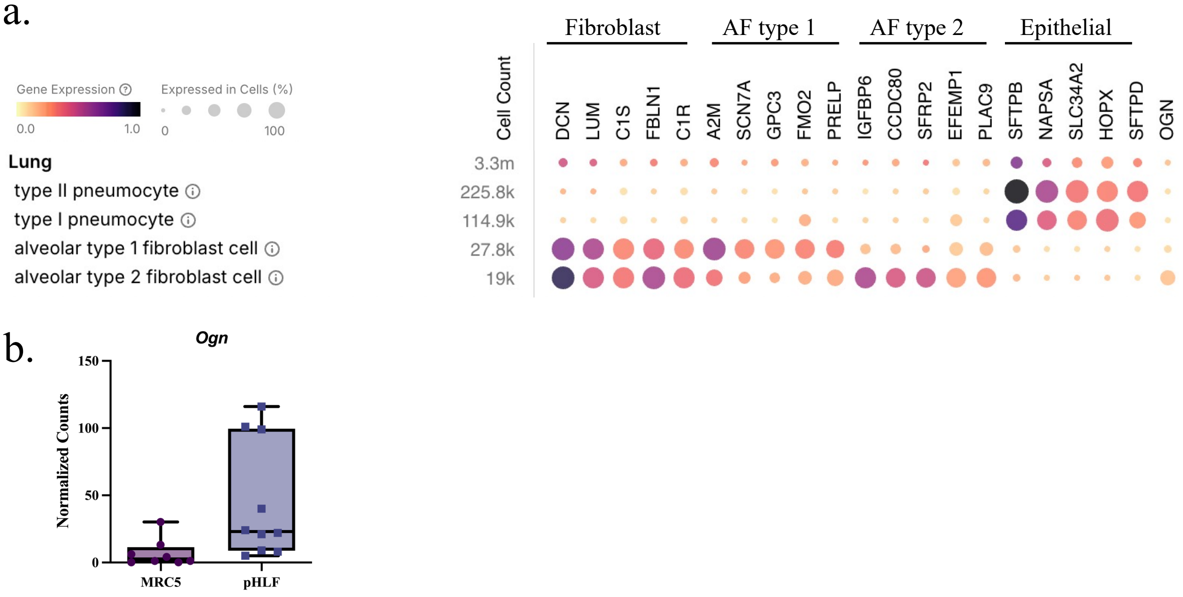

Fig. S3. OGN expression in lung fibroblasts.

a The expression of markers for fibroblasts (*DCN*, *LUM*, *C1S*, *FBLN1,* and *C1R*), alveolar fibroblast (AF) type 1 (*SCN7A*, *GPC3*, *FMO2*, *PRELP*), AF type 2 (*IGFBP6*, *CCDC80*, *SFRP2*, *EFEMP1*, *PLAC9*), epithelial cells (*SFTPB*, *NAPSA*, *SLC34A2*, *HOPX*, and *SFTPD*), and *OGN*. Data were extracted from a public scRNA-Seq dataset (https://lungmap.nl/). **b** Normalized counts of *OGN* in MRC5 fibroblasts and pulmonary human lung fibroblasts (pHLF).

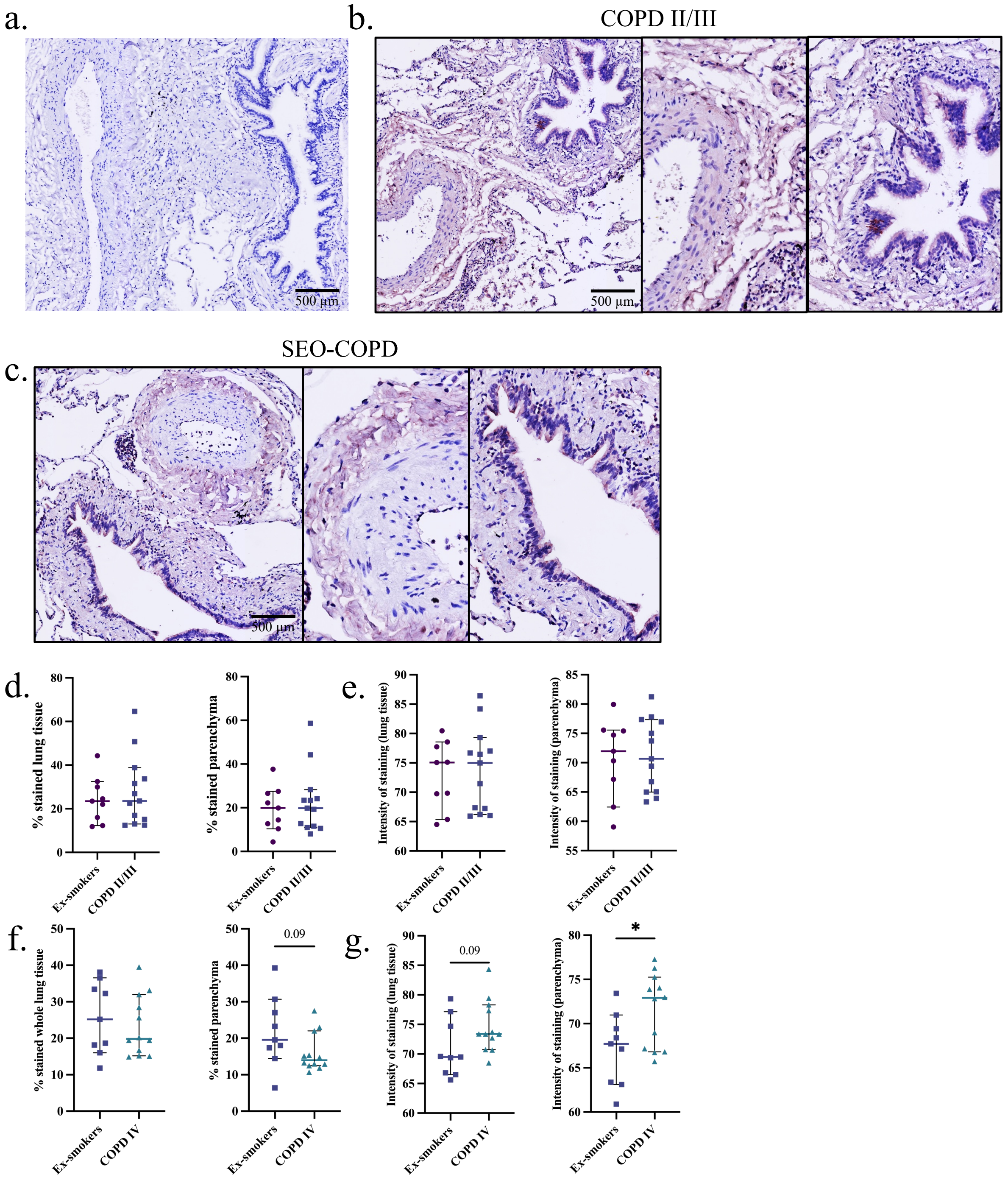

Fig. S4. OGN expression in moderate-severe COPD lung tissue.

a Example image of negative control staining in human whole lung tissue. **b-c** Example image of OGN staining in whole lung tissue in moderate-severe COPD (II/III) and SEO-COPD (scale = 500 µm). **d** Positively stained area percentage (%) for OGN in whole lung tissue and parenchyma in COPD II/III patients. **e** Intensity of staining for OGN in whole lung tissue and parenchyma in COPD II/III patients. **f** Positively stained area percentage (%) for OGN in whole lung tissue and parenchyma in SEO-COPD

patients. **g** OGN staining intensity in whole lung tissue and parenchyma in SEO-COPD patients. Statistically significant comparisons are represented by *$p < 0.05$.

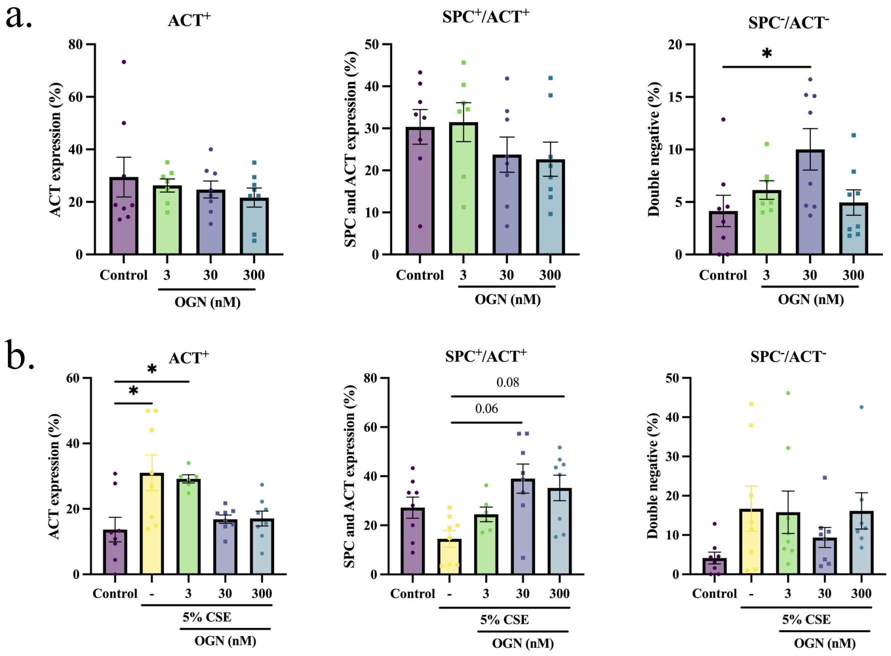

Fig S5. Osteoglycin supports organoid formation and differentiation.

a Immunohistochemistry quantification for ACT^+, SPC^+/ACT^+, and SPC^-/ACT^- organoids (mean ± SEM, N=8, paired one-way ANOVA with Tukey test for multiple testing). **b** Immunohistochemistry quantification for ACT^+, SPC^+/ACT^+, and SPC^-/ACT^- organoids in the presence of CSE (mean ± SEM, N=8-11, paired one-way ANOVA with Tukey test for multiple testing). Statistically significant comparisons are represented by $*p < 0.05$.

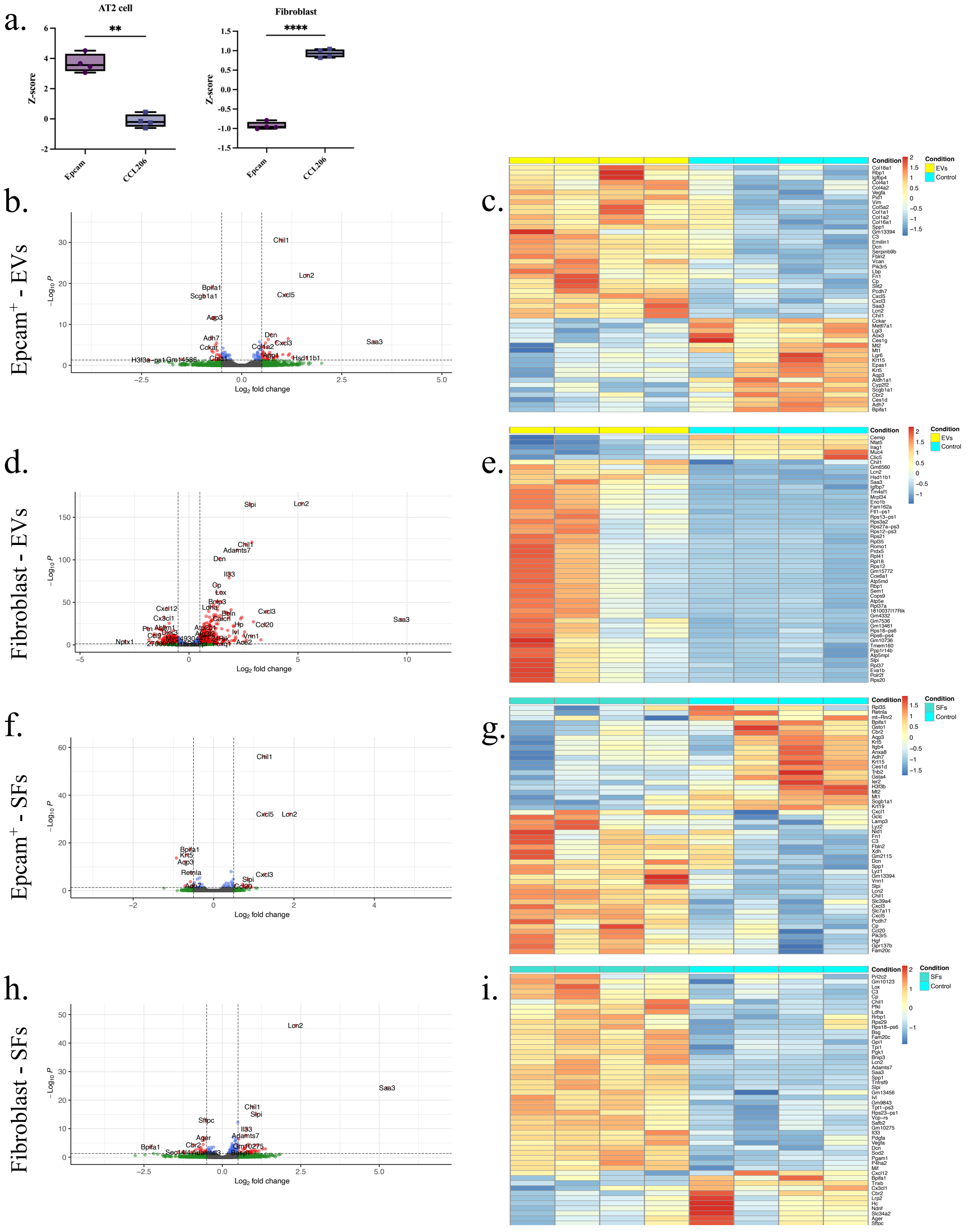

Fig. S6. EVs and SFs induce transcriptional changes in Epcam⁺ cells and fibroblasts.

a AT2 cell and fibroblast signature within control Epcam⁺ cell and fibroblast population (mean ± min/max, N=4, paired two-tailed T-test). **b** Volcano plot illustrating the response to EVs versus control in epithelial progenitor cells (cut-offs: padj<0.05 and logFold>1). **c** Heatmap with the top 50 significant

genes up- or downregulated in epithelial progenitor cells with EVs. **d** Volcano plot illustrating the response to EVs versus control in fibroblasts (cut-offs: padj<0.05 and logFold>1). **e** Heatmap with the top 50 significant genes up- or downregulated in fibroblasts treated with EVs. **f** Volcano plot illustrating the response to SFs versus control in epithelial progenitor cells (cut-offs: padj<0.05 and logFold>1). **g** Heatmap with the top 50 significant genes up- or downregulated in epithelial progenitor cells with SFs. **h** Volcano plot illustrating the response to SFs versus control in fibroblasts (cut-offs: padj<0.05 and logFold>1). **i** Heatmap with the top 50 significant genes up- or downregulated in fibroblasts treated with SFs. Statistically significant comparisons are represented by **p < 0.01 and ****p < 0.0001.

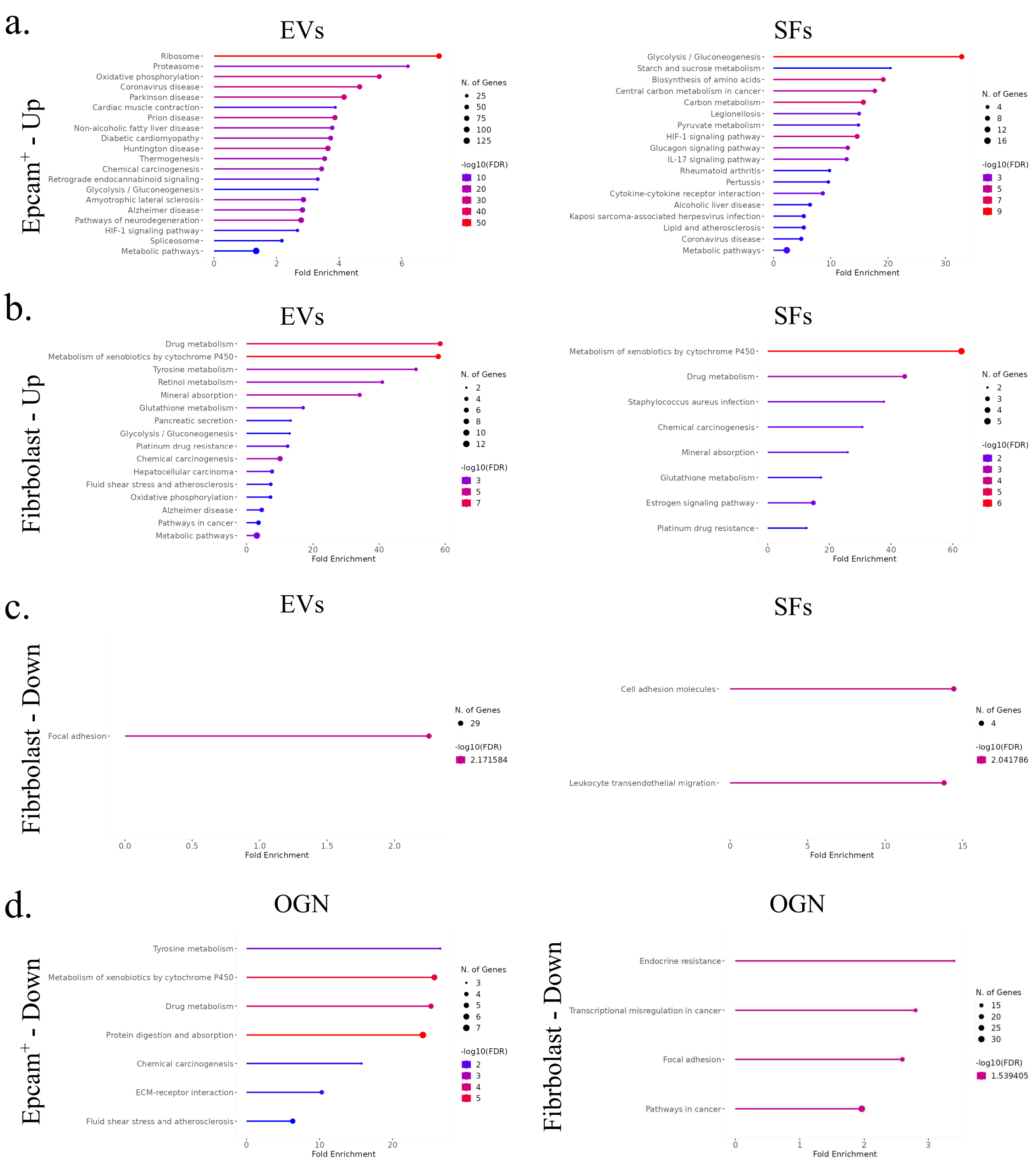

Fig. S7. Gene set enrichment analysis in Epcam⁺ cells and fibroblasts upon treatment with EVs, SFs, or OGN.

a The top upregulated pathways in epithelial progenitors treated with EVs or SFs. **b** The top upregulated pathways in fibroblasts treated with EVs or SFs. **c** The top-downregulated pathway(s) in fibroblasts treated with EVs or SFs. **d** The top downregulated pathways in epithelial progenitors or fibroblasts treated with OGN.

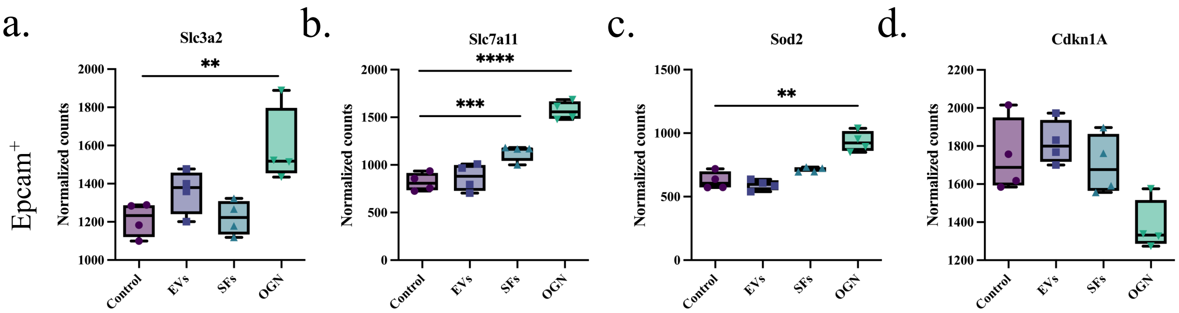

Fig. S8. Osteoglycin increases the expression of protective genes in epithelial cells.

a-d Normalized counts of heavy chain subunit of cystine and glutamine anti-transporter (*Slc3a2*), light chain subunit of cystine and glutamine anti-transporter (*Slc7a11*), superoxide dismutase 2 (*Sod2*), and senescence marker (*Cdkn1a*) upon treatment of epithelial progenitors with EVs, SFs, or OGN (mean ± min/max, N=4, paired one-way ANOVA with Tukey test for multiple testing). Statistically significant comparisons are represented by **p < 0.01, ***p < 0.001, and ****p < 0.0001.

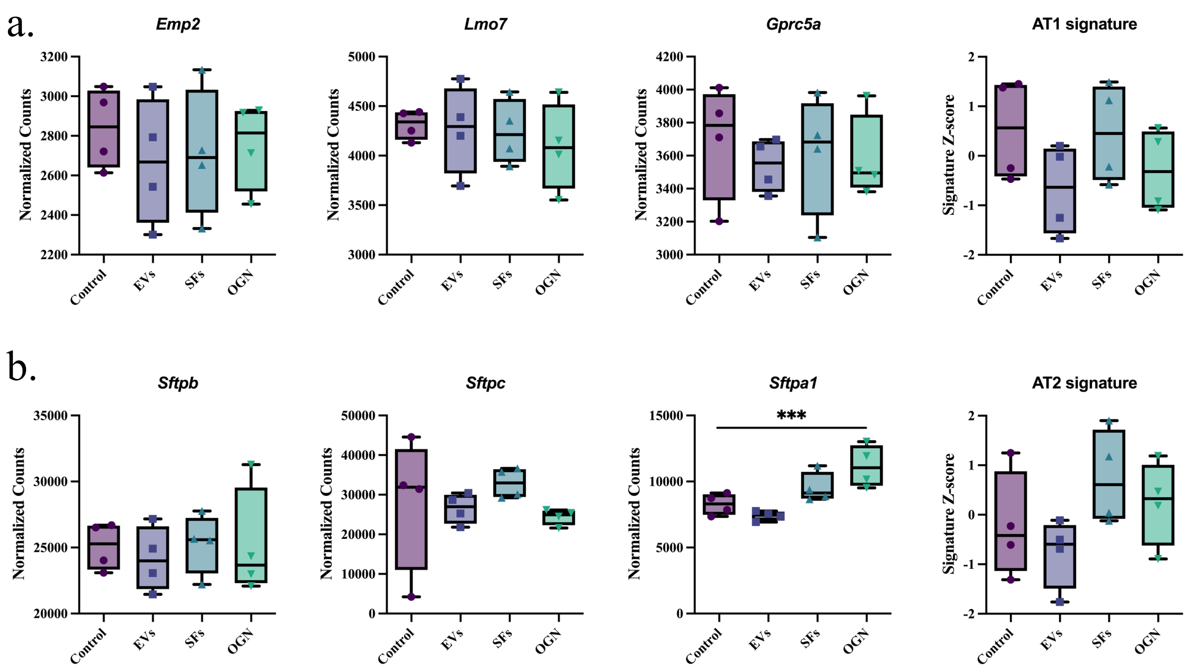

Fig. S9. Osteoglycin increases the expression of protective genes in epithelial cells.

a Normalized counts of markers for alveolar type 1 (AT1) cells (*Emp2*, *Lmo7*, and *Gprc5a*) and composite AT1 gene signature based on the top 10 AT1 marker genes upon treatment of epithelial progenitor cells with EVs, SFs, or OGN (mean ± min/max, N=4, paired one-way ANOVA with Tukey test for multiple testing). **b** Normalized counts of markers for alveolar type 2 (AT2) cells (*Sftpb*, *Sftpc*, and *Sftpa1*) and composite AT2 gene signature based on the top 10 AT2 marker genes upon treatment of epithelial progenitor cells with EVs, SFs, or OGN (mean ± min/max, N=4, paired one-way ANOVA with Tukey test for multiple testing). Statistically significant comparisons are represented by ***$p < 0.001$.

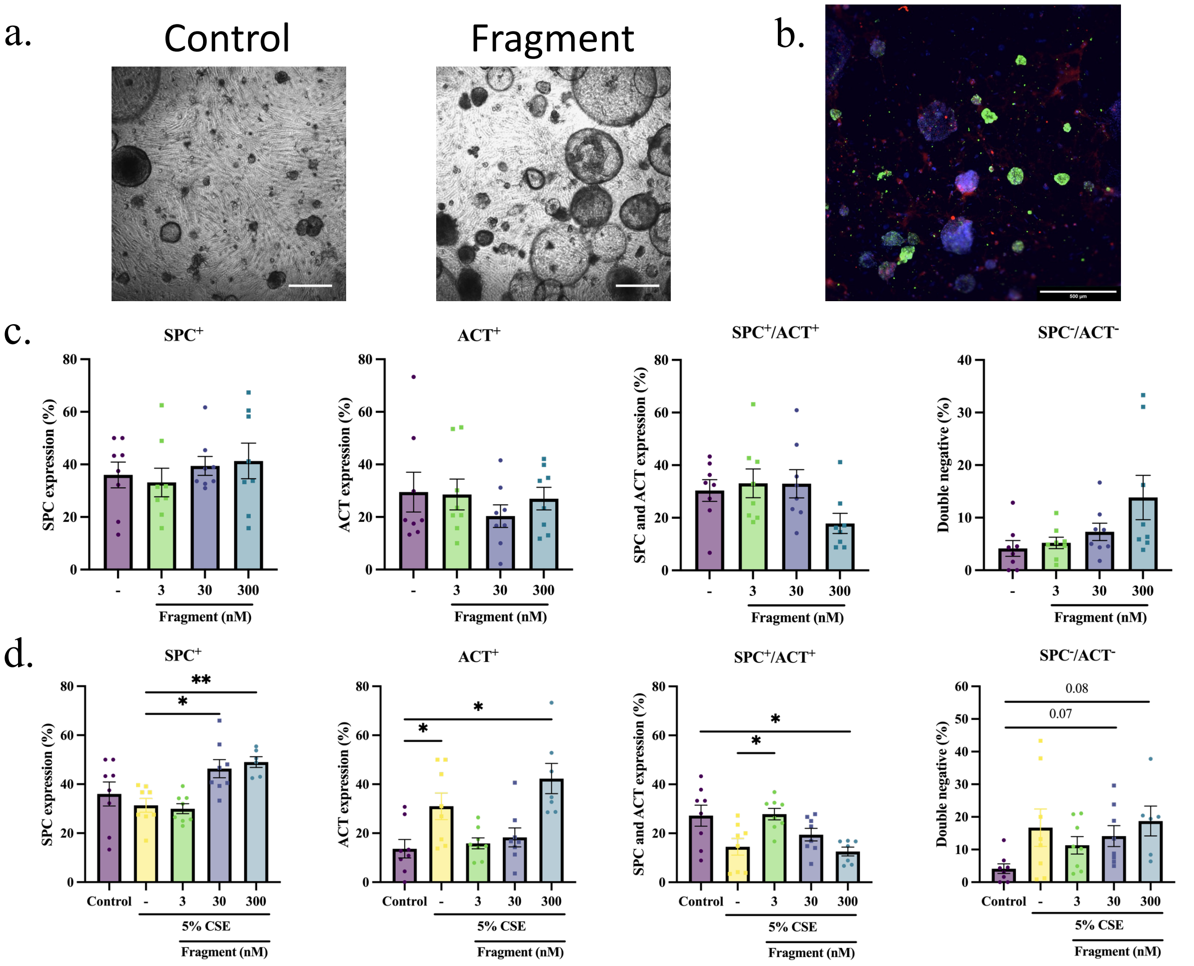

Fig. S10. The active fragment of osteoglycin induces alveolar organoid formation.

a Representative brightfield images of murine lung organoids (scale = 500 µm). **b** Representative immunofluorescence image of stained organoids treated with OGN fragment for airway-type organoids (acetylated α-tubulin, red), alveolar-type organoids (surfactant protein C, green), and Dapi (nuclei, blue) (scale = 500 µm). **c** Immunohistochemistry quantification for SPC^+, ACT^+, SPC^+/ACT^+, and SPC^-/ACT^- organoids (mean ± SEM, N=8, paired one-way ANOVA with Tukey test for multiple testing). **d** Immunohistochemistry quantification for SPC^+, ACT^+, SPC^+/ACT^+, and SPC^-/ACT^- organoids in the presence of CSE (mean ± SEM, N=8, paired one-way ANOVA with Tukey test for multiple testing). Statistically significant comparisons are represented by $^*p < 0.05$ and $^{**}p < 0.01$.

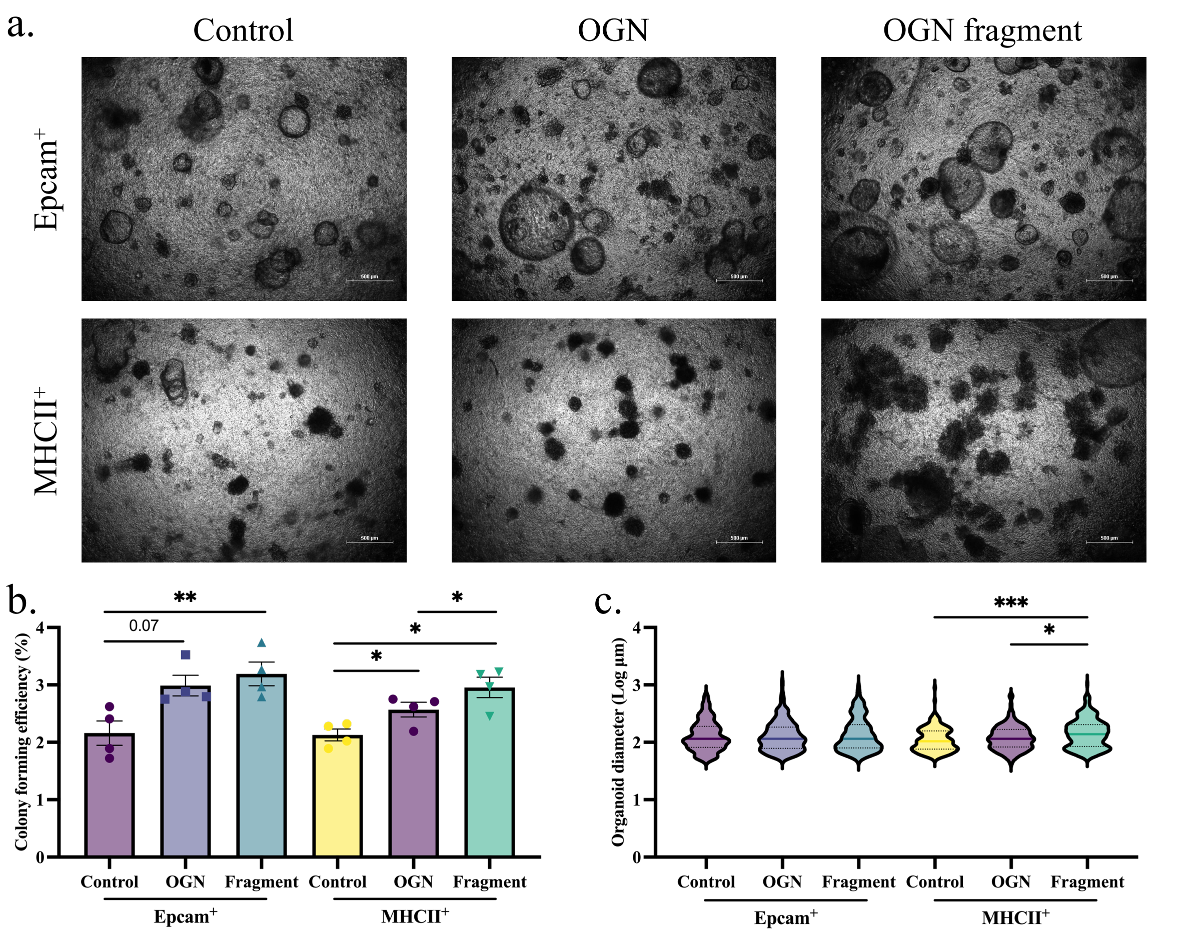

Fig. S11. Osteoglycin and its active fragment induce organoid formation from MHCII+ and Epcam+ cells. a Representative brightfield images of murine lung organoids from Epcam+ cells and MHCII+ cells (scale = 500 µm). **b** Colony forming efficiency of murine Epam+ and MHC+ cultures treated with OGN or its active fragment on day 14 (mean ± SEM, N=6, paired Friedman test). **c** Log of organoid diameter of murine Epam+ and MHC+ cultures treated with OGN or its active fragment on day 14 (median is shown, N=6, Kolmogorov-Smirnov test (after Bonferroni correction: α = 0.025)). Statistically significant comparisons are represented by *p < 0.05, **p < 0.01, and ***p < 0.001.

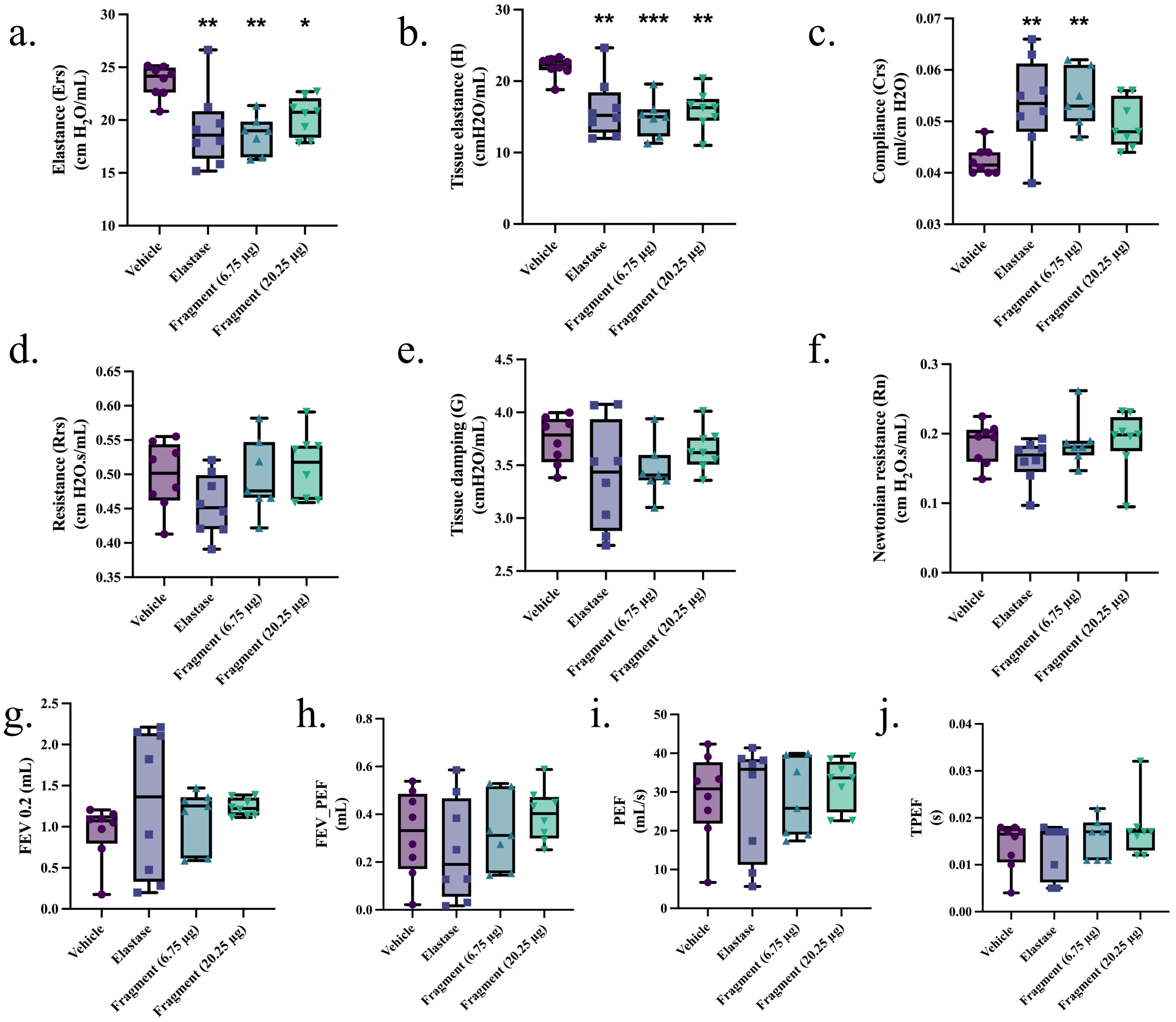

Fig. S12. Lung tissue characteristics and lung function parameters in murine elastase-induced lung injury model.

a-f Lung tissue characteristics: elastance, tissue elastance, compliance, resistance, tissue damping, and Newtonian resistance as measured with the FlexiVent (median ± min/max data point, N=7-8, One-Way ANOVA followed by Sidak's multiple comparison). **g-j** Forced expiratory volume at 0.2 seconds (FEV0.2), forced expiratory volume at peak expiratory flow (FEV_PEF), peak expiratory flow (PEF), time to reach peak expiratory flow (TPEF) as measured with the FlexiVent (median ± min/max data point, N=7-8, One-Way ANOVA followed by Sidak's multiple comparison). Statistically significant comparisons are represented by $^*p < 0.05$, $^{**}p < 0.01$, and $^{***}p < 0.001$.

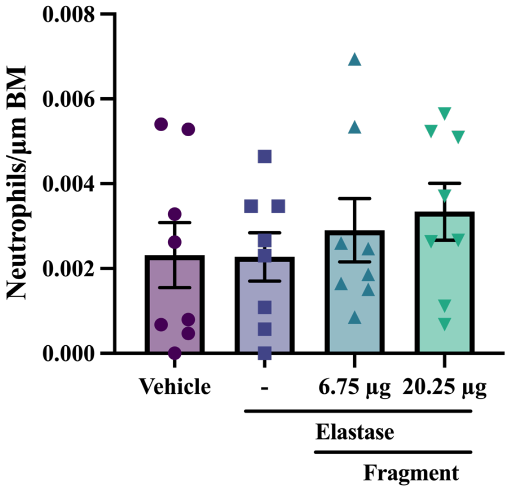

Fig. S13. Neutrophil infiltration in murine elastase-induced lung injury model. Neutrophil infiltration as determined by H&E staining in lung tissue from murine elastase-induced lung injury model (mean ± SEM, N=8, One-Way ANOVA followed by Sidak's multiple comparison).

Supplementary Data:

Supplementary Data 1. Assessment of proteomics analysis based on MISEV guidelines.

Data are categorized based on the MISEV 2018 guidelines. Green-colored rows represent higher relative abundance in EV samples, blue rows represent higher relative abundance in SF samples, and red rows represent ratios that could not be calculated. N/A, not applicable.

Supplementary Data 2. Relative abundance of proteins present in all three replicates of lung fibroblast-derived EVs and SFs.

Data are organized alphabetically.

Supplementary Data 3. Detailed proteomics-guided drug discovery strategy.

Factors are present in all biological replicates (N=3) of both EVs and SFs. N/A is not applicable.

Supplementary Data 4. Information of recombinant proteins screened in organoid assay.

Supplementary Data 5A. Patient characteristics of immunohistochemistry OGN staining in control tissue.

The FEV_1 and FVC data were not available for lung tissue from Rochester (N=7) and for a few cases from Groningen (N=5). Data is presented as the median with the range between brackets. $FEV_{1\%pred}$ = highest measurement of forced expiratory volume in 1s for an individual; FVC = forced vital capacity; NA = not applicable; * = a significant difference in the number of pack years between current and ex-smokers (unpaired two-tailed T-test).

Supplementary Data 5B. Patient characteristics for immunohistochemistry OGN staining in COPD tissue.

SEO-COPD (N=12) and moderate-severe COPD (n=14) were matched in terms of age, sex, and smoking status to respective control groups. Data is presented as the median with the range between brackets. $FEV_{1\%pred}$ = highest measurement of forced expiratory volume in 1s for an individual; FVC = forced vital capacity; NA = not applicable. Statistical significance was determined with an unpaired two-tailed T-test; p-values <0.05 were considered significant.

Supplementary Data 6. Overview of the genes included for the gene signatures of alveolar type II cells and fibroblasts

Supplementary Data 7. Patient characteristics of lung tissue from COPD IV patients used for organoid culture

The FEV_1 and FVC data were unavailable for lung tissue from one donor patient. Data is presented as the median with the range between brackets. FEV1 = highest measured forced expiratory volume in 1s for an individual after bronchodilation (BD); FVC = forced vital capacity.
